# Metabarcoding of marine environmental DNA based on mitochondrial and nuclear genes

**DOI:** 10.1038/s41598-018-32917-x

**Published:** 2018-10-04

**Authors:** Babett Günther, Thomas Knebelsberger, Hermann Neumann, Silke Laakmann, Pedro Martínez Arbizu

**Affiliations:** 1 Senckenberg am Meer, German Centre for Marine Biodiversity Research, Südstrand 44, 26382 Wilhelmshaven, Germany; 2 Present Address: Ifremer (Institut français de recherche pour l’exploitation de la mer), Avenue Jean Monnet BP 171, 34200 Sète, France; 3 Senckenberg am Meer, Marine Research, Südstrand 40, 26382 Wilhelmshaven, Germany; 4 Present Address: Helmholtz Institute for Functional Marine Biodiversity at the University of Oldenburg (HIFMB), Ammerländer Heerstraße 231, 26129 Oldenburg, Germany

**Keywords:** Biodiversity, Molecular ecology, Marine biology

## Abstract

We establish the new approach of environmental DNA (eDNA) analyses for the North Sea. Our study uses a multigene approach, including the mitochondrial cytochrome-*c*-oxidase subunit I (COI) gene for analyzing species composition and the nuclear hypervariable region V8 of 18S rDNA for analyzing supraspecific biodiversity. A new minibarcode primer (124 bp) was created on the basis of a metazoan COI barcode library with 506 species and tested *in silico*, *in vitro*, and *in situ*. We applied high throughput sequencing to filtrates of 23 near-bottom water samples taken at three seasons from 14 stations. The set of COI primers allowed amplification of mitochondrial minibarcodes for diverse metazoan phyla and the differentiation at the species level for more than 99% of the specimens in the dataset. Our results revealed that the number of sequences is not consistent with proportions in the given DNA mixture. Altogether, environmental sequences could be assigned to 114 species and to 12 metazoan phyla. A spatial distribution of taxa recovered by eDNA was congruent with known distributions. Finally, the successful detection of species and biodiversity depends on a comprehensive sequence reference database. Our study offers a powerful tool for future biodiversity research, including the detection of nonnative species.

## Introduction

Molecular-DNA-based species-identification methods like DNA barcoding have been established during recent decades for the study of biodiversity^1–4^. In addition, relatively new approaches like metabarcoding of environmental DNA (eDNA) have been tested^5,6^. eDNA is defined as the whole DNA extracted from environmental samples as soil, water, or air^7,8^. However, it is to distinguish between intraorganismal, extraorganismal and extramembranous DNA within an eDNA sample^9^. This study is focusing on “extracellular” or “extraorganismal” eDNA present in the marine environment^5^. The first analyses of microbial eDNA from sediments were made already in the last Century^10,11^. In the last decade, analyses of eDNA have been generalized to studies of flora and fauna. Studies^12,13^ have already outlined various applications of eDNA to describing ecosystem processes, estimating relative abundance, and detecting known and unknown invasive species. They have even been used to monitor present and ancient environments^14^. Studies have also revealed a high comparability between eDNA approaches and established survey methods, especially for fish (see, e.g.^15,16^). For these reasons, eDNA analysis is a promising emerging tool for species detection in the context of routine monitoring programs.

Until now, only a few studies applied eDNA analysis to identification of species from marine water samples or seawater aquarium tanks. Most studies have focused only on single taxonomic groups like fish^15,17–20^, mammals^21^, or all vertebrates^22^. In contrast, O’Donnell^23^ used this approach for all metazoans, in order to quantify the spatial patterns of communities for a dynamic marine environment. Altogether, the length of the DNA fragments analyzed ranged from 60 to 185 base pairs (bp) mostly from mitochondrial genes like ribosomal 12S or 16S or cytochrome b (cyt*b*). One advantage of mitochondrial genes is that the results have been shown to correlate with rank abundance in some cases^16,24,25^.

Several studies of marine metagenomics have used the hypervariable regions of 18S, like V1–V2^26^, V4^27,28^, or V7^24^. The V8 region was chosen for our study on the basis of its low amplicon-length variability^29^. 18S is known to underestimate the number of metazoan species^30^, but primer sites are highly conserved in 18S rDNA and permit amplification of a broad range of metazoan taxa. Besides 18S and cyt*b*, DNA barcoding has become more and more important for molecular species identification during the last decade. This approach is based on the analyses of a ~650-bp fragment of the cytochrome *c* oxidase subunit I (COI) mitochondrial gene region^3,4,30^.

Moreover, universal and taxon-specific COI minibarcodes (ranging between ~100 and 400 bp) have been developed in recent years for analysis of degraded DNA in marine systems or gut contents^31,32^. In addition, Shokralla^33^ reviewed the possible advantages of minibarcodes for the application of eDNA analysis resulting from its species-level identification for broad ranges of metazoans.

For taxonomic validation of eDNA analysis results, a reliable DNA sequence reference library is essential. From 2010 through 2016, a DNA barcode (COI) reference library was produced for North Sea metazoans at the Senckenberg Institute in Wilhelmshaven^1,2,34–43^. Species material, including the morphological species description came from the Long-term Ecological Research (LTER) sites as “North Sea Benthos Observatory” and the “Helgoland Roads” (for more information see: https://deims.org/network/germany-lter-d and https://deims.org/1e96ef9b-0915-4661-849f-b3a72f5aa9b1), ensuring high taxonomic quality of that DNA reference library.

The aim of our study was to test the efficiency of metazoan species detection and community composition in the German Bight, North Sea, by means of extraorganismal eDNA metabarcoding of water samples from different sampling locations in different seasons. Our approach combines and integrates state-of-the-art metabarcoding by high-throughput sequencing technologies, eDNA analyses, and minibarcoding. In this approach we test the efficiency of mitochondrial minibarcodes (COI) and nuclear 18S rDNA metabarcoding *in silico* (bioinformatically), *in vitro* (in extracted DNA tissue samples), and *in situ* (in artificial mixed DNA and eDNA samples).

## Results

### Primer efficiency for species detection

#### In silico

The COI primer pair we designed (Table 1) and tested for amplification performance in the *in silico* analysis. In the alignment including the consensus sequences of 506 species, primer binding with up to four mismatches was not possible for only 14 species (2.8%). In only 122 specimens out of 3,674 (3.3%) could the primers not be assembled. In particular, the forward primer fit very well; when three possible mismatches were allowed only four specimens (two species) were not amplifiable. Moreover, even within the short fragment of 124 bp, the differentiation between species with at least 2% dissimilarity was possible for more than 99% of the species. Only in two cases closely related species could not be differentiated with the full barcode and thus not with minibarcodes (*Tomopteris cf*. helgolandicus/ *cf*. *septentrionalis*; *Lanice conchilega* and *Pista cristata*).

**Table 1:** PCR primers, including their tags for sample pooling.

Primer name	Direc-tion	Sequence 5′-3	Reference
COI			
mlCOIintR	R	GGRGGRTASACSGTTCASCCSGTSCC	Leray *et al*.^31^
mlCOIintK	R	GGRGGRTAWACWGTTCAWCCWGTWCC	Present study
nsCOIFo	F	THATRATNGGNGGNTTYGGNAAHTG	Present study
Tags	F	actgac, gtagca, gacagt, tgtacg, catctg	Present study
Tags	R	gacgta, tctgag, gctagt, agcact, agagac	Present study
18S			
18S#4	F	AGGTCWGTRATGCCCTYMG	Machida & Knowlton 2012^29^
18S#5_RC	R	TGYACAAAGGBCAGGGAC C	Machida & Knowlton 2012^29^
Tags	F	gtca, catg, acgt, tagc, agac	Present study
Tags	R	actc, gaga, ctct, gcat, tgag	Present study

#### In vitro

The results of our *in vitro* tests of both primer pairs in single PCRs of 30 targets and 15 nontarget DNA extracts are given in Supplementary Information 1. For all target specimens, PCR products using minibarcodes could be amplified, even for one specimen (*Aglantha digitale*) with five mismatches in the reverse and three in the forward direction. In addition, PCR products of single-cell eukaryotes and diatoms were observed. For 18S, besides all nontargets, all target specimens and species could be amplified. Therefore, nonmetazoans could only be excluded from results by bioinformatic analyses after the sequencing process.

#### In situ

In the artificial sample from the Illumina output of COI, all 30 species were detected with 135 to 66,610 sequences and 1–19 OTUs per specimen (Supplementary Information 2). But for 18S analyses, not all species could be redetected, or with 3 to 62,928 sequences and up to 49 OTUs per specimen. For both amphipods, as well as the mysid *Mesopodopsis slabberi*, the 18S sequences were too long (>180 bp) to be detected with the 125-bp paired-end reading length. For the decapods *Liocarcinus holsatus* and *Cancer pagurus*, the sequences are identical and therefore could not be separated. At least three species were not detected— the crustaceans *Evadne spinifera* and *Balanus crenatus* and the squid *Loligo forbesii—*so even if all specimens could be amplified by PCR, not all could be detected after Illumina sequencing.

**Figure 1:**
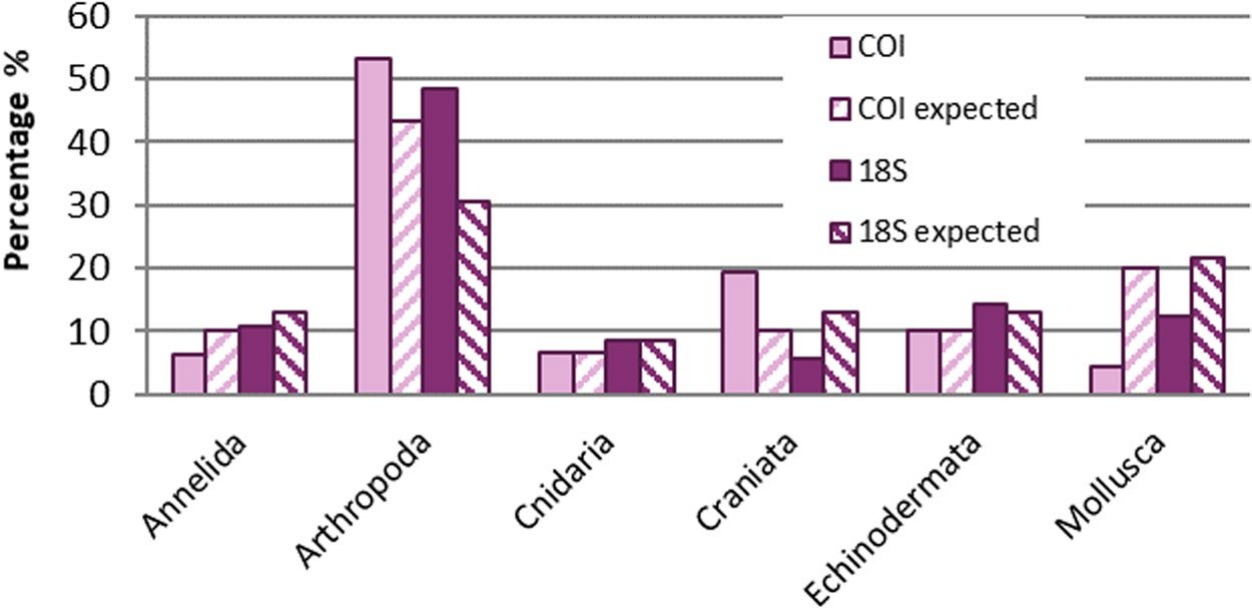
Expected and detected sequence counts (as percentages) of target specimens (at the level of phyla) in the artificial sample for cytochrome *c* oxidase subunit I (COI) and nuclear hypervariable region V8 of 18S rDNA samples. Percentages are calculated by taxon reads to all identified reads per gene region. Expected reads calculated based on equalized ngDNA amount, with taxa excluded for 18S rDNA which cannot be sequenced in the 125-bp paired-end reading length. Prepared by Babett Günther using Microsoft Excel 2010 (www.microsoft.com).

When we compared 18S rDNA and COI analyses of the artificial sample, the number of sequences for one specimen ranged from less than 1,000 to more than 66,000, as did the numbers of OTUs (Supplementary Information 2). The expected and detected percentages of sequences at the phylum level are shown in Fig. 1. At least for the Cnidaria and Echinodermata, detection is similar for the two gene regions; divergences ranged from 0.02 to 1.30%. In contrast, the arthropods, mollusks, and Craniata differed markedly; divergences ranged from 7 to 18%.

Our results revealed that neither number of sequences nor their proportions at the phylum level nor the number of OTUs per specimen is consistent with proportions in the given DNA mixture. This result leads to the suggestion that for environmental samples, only presence-absence data or the relative variation among samples can be analyzed, separately by gene regions.

### Species identification by COI

**Figure 2:**
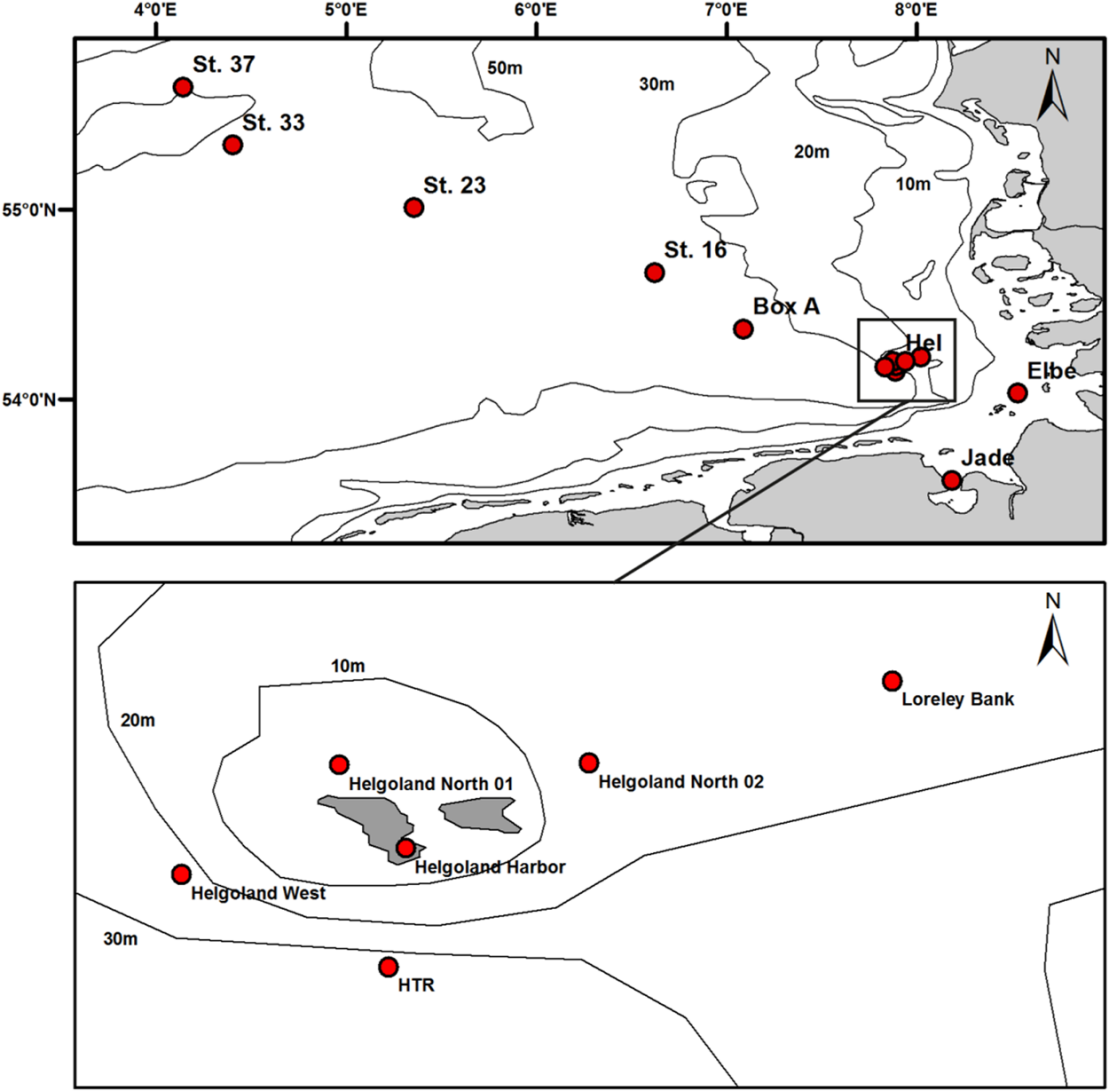
Sampling sites in the German Bight, North Sea. St., station while Elbe “indicating the Elbe estuary, “Jade” the Jade Bight, a station in front of the Jade Weser Port. Stations around Helgoland (Hel) are shown in greater detail, here HTR, “Helgoländer Tiefe Rinne” (trench in front of Helgoland); Loreley, Loreley Bank; HeN1, Helgoland North 01; HeN2, Helgoland North 02; HeW, Helgoland West. Prepared by Hermann Neumann using ArcMap 10.4.1 (www.esri.de) and processed by Adobe Illustrator CS6 (www.adobe.com).

**Figure 3:**
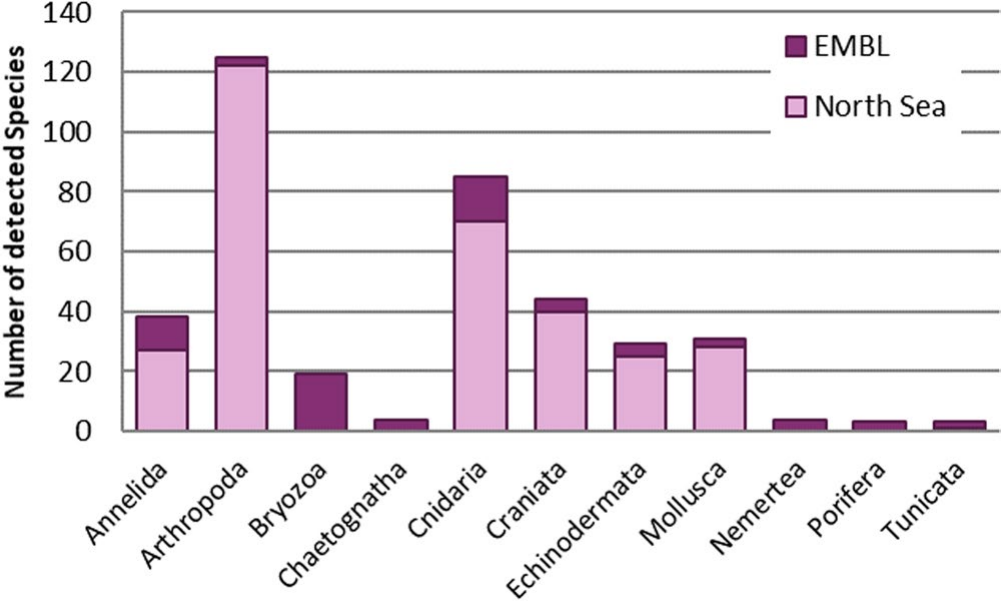
Number of species detected with COI (per phylum) from all 20 environmental samples with identified species. The Senckenberg Barcode reference library (North Sea) and European Molecular Biology Laboratory (EMBL) database were used for assignment. Prepared by Babett Günther using Microsoft Excel 2010 (www.microsoft.com).

Species lists based on the results of assignment from environmental samples within the Senckenberg Barcode reference library and COI EMBL reference database are given in Supplementary Information 3. No species were found in samples 4 and 6 (HTR, Jade-JWP see Fig. 2) for both spring samples and one summer sample (8, Station 33). The variation in phyla detected is displayed in Fig. 3, which shows the detection for the 11 phyla Annelida, Arthropoda, Bryozoa, Chaetognatha, Cnidaria, Craniata, Echinodermata, Mollusca, Nemertea, Porifera, and Tunicata, but differences exist in the detection assignment of the sequences to the Senckenberg Barcode reference library and the COI EMBL reference database.

On the basis of the Senckenberg Barcode reference library, up to 45 species per sample were detected, and 313 were detected for all environmental samples together (Supplementary Information 3). In general, more species were detected in samples from later seasons and in samples from around Helgoland. In total, 82 of the 506 species in the Senckenberg Barcode reference library were detected.

Through additional taxonomic assignment with the COI EMBL reference database, 32 additional species and, overall, 72 detections were identified (Supplementary Information 3). In most cases, the species found or at least their genera are known to occur at these sampling sites. Within this assignment, two terrestrial species, *Sus scrofa* (wild boar) and *Rhopalosiphum padi* (aphid), were recorded but have been excluded from the amount of detected species and the following analyses. Species from Bryozoa, Chaetognatha, Porifera, and Nemertea could be detected by the assignment with COI EMBL reference data base only, because their sequence data do not exist in the Senckenberg Barcode reference library.

Most species detected were crustaceans, cnidarians, and craniata (all fish species) (see Fig. 3). In these groups we also found the most abundant species—like *Aurelia aurita* and *Eucheilota maculata* (Cnidaria), *Balanus balanus* and *Temora longicornis* (Crustacea), and *Pleuronectes platessa* (Pisces)—each in at least 10 samples.

### 18S detections

The relative abundances of the assigned 18S rDNA sequences from the environmental samples were determined. For every sample, 1,633 to 121,717 sequences could be assigned to one of the nine phyla Annelida, Arthropoda, Bryozoa, Cnidaria, Craniata, Ctenophora, Echinodermata, Mollusca, and Tunicata.

**Figure 4:**
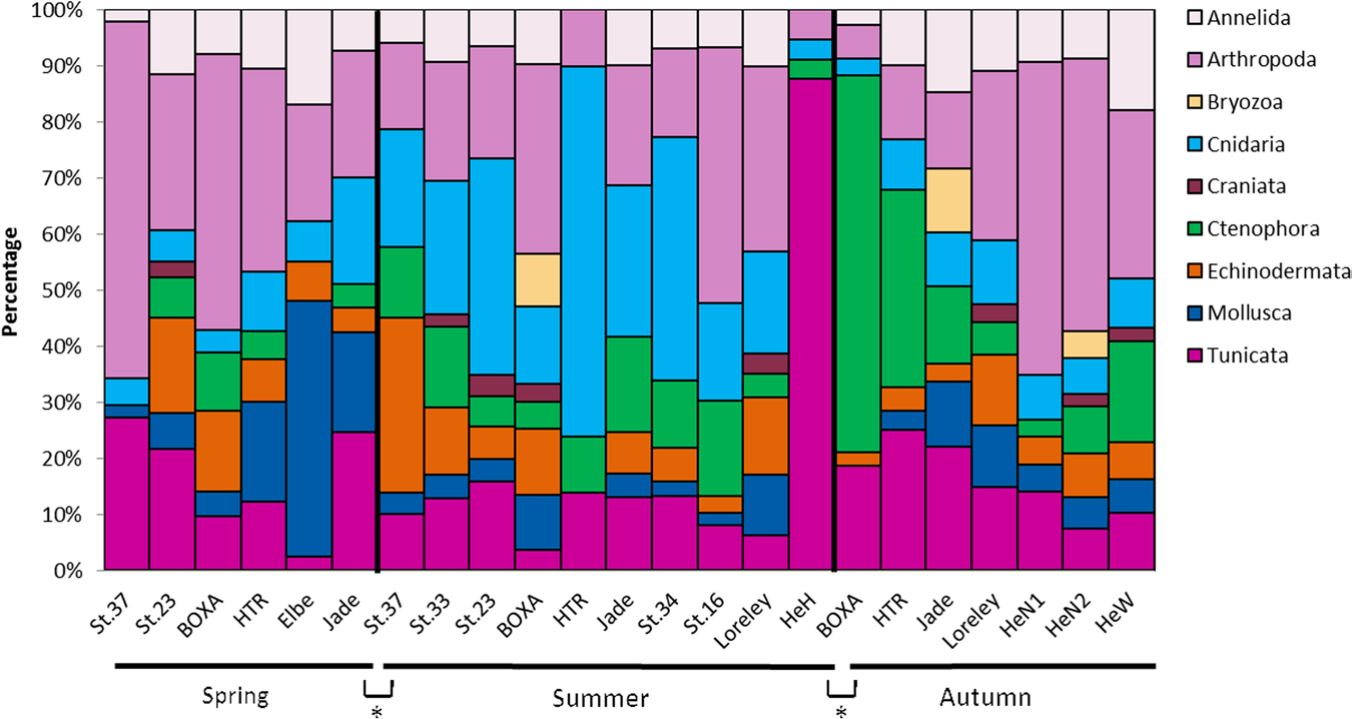
Relative abundances of phyla based on 18S rDNA analyses. Percentages are of the overall number of sequences within one sample. Following abbreviations indicate stations: HTR, “Helgoländer Tiefe Rinne”; Loreley, Loreley Bank; HeN1 Helgoland North 01; HeN2, Helgoland North 02; HeW, Helgoland West; HeH, Helgoland Harbor. The * indicating the detailed significant differences between spring (p = 0.02) and autumn (p = 0.02) with summer (PREMANOVA pair-wise test). Prepared by Babett Günther using Microsoft Excel 2010 (www.microsoft.com).

The relative abundances of the phyla are shown in Fig. 4. Some samples were dominated by one phylum: the Elbe estuary in spring by Mollusca, HTR (“Helgoländer Tiefe Rinne”) in summer by Cnidaria, Helgoland Harbor in summer by Tunicata, Box A in autumn by Ctenophora, St. 37 in spring and HeN1 in autumn by Arthropoda (Fig. 4). In summer, Cnidaria dominated the relative abundances, whereas in autumn, especially in the west of Helgoland, more Ctenophora than Cnidaria occurred.

### Comparing 18S and COI output

**Figure 5:**
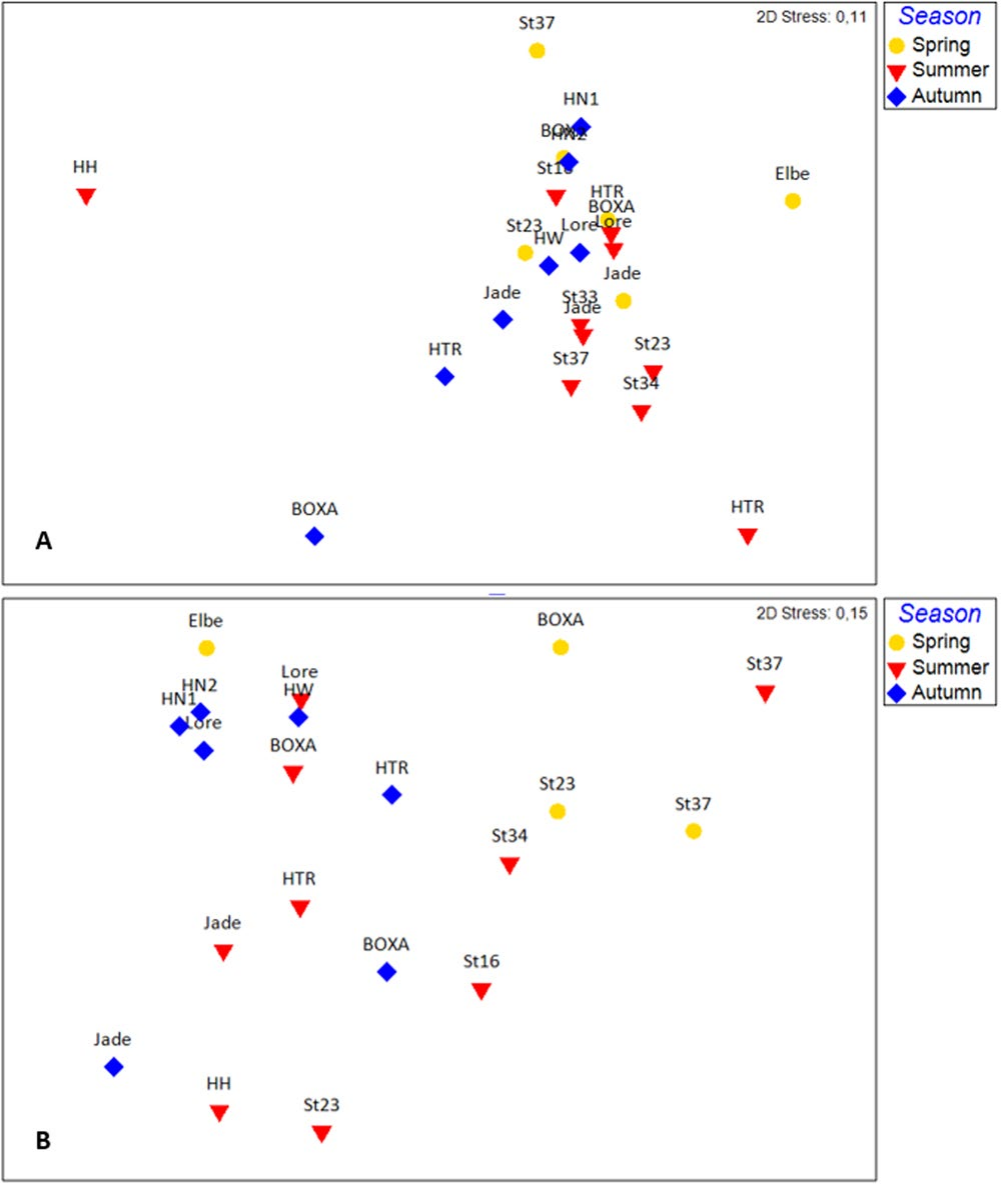
Non-metric multidimensional scaling (nMDS) plots visualize similarities of samples for seasonality. Analyses based for (**A**) on Euclidean distance of relative abundances of phyla (18S rDNA) and (**B**) on presence-absence Sørensen similarity (COI) for species composition. Following abbreviations indicate stations: HTR, “Helgoländer Tiefe Rinne”; Loreley, Loreley Bank; HN1 Helgoland North 01; HN2, Helgoland North 02; HW, Helgoland West; HH, Helgoland Harbor. Prepared by Babett Günther using PRIMER 6 (www.primer-e.com).

After the homogeneity of sample dispersions was confirmed (PERMDISP, both p > 0.2), seasonality was analyzed (Fig. 5). For both gene regions, distance analyses revealed season to be a significant factor (PREMANOVA, COI, p = 0.03; 18S, p = 0.01). The pair-wise test revealed summer to differ significantly from both spring (p = 0.02) and autumn (p = 0.02) for 18S rDNA analyses. In the species analyses (COI), only the spring and autumn samples differed significantly (p = 0.04).

Only occasionally, consensus in between 18S rDNA and COI analyses in phylum and species detection could be demonstrated. For example, at Helgoland Harbor in summer, 18S analysis detected high relative abundance of Tunicata, whereas COI analyses confirmed the occurrence of the tunicate species *Ascidiella aspersa*, which showed the highest number of sequences in this area. Also at the station HTR, Cnidaria dominated the relative abundance in 18S DNA, and COI confirmed nine cnidarian species in a total of 14 species detected. In contrast, at the Elbe estuary in spring, we found a high percentage of mollusks in 18S rDNA analyses, but only one squid species (5.3% of identified reads) was confirmed by COI.

**Figure 6:**
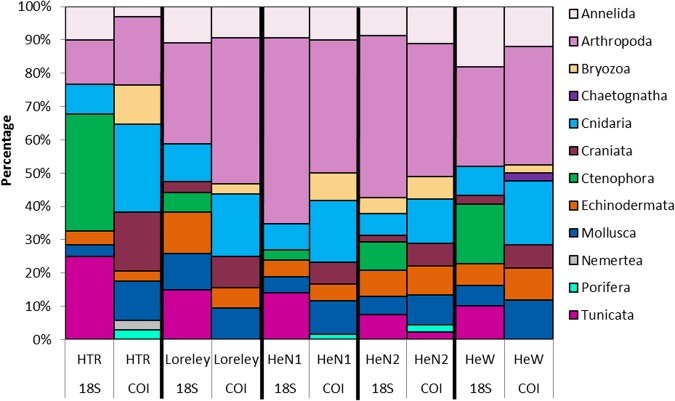
Locations of fall sampling around Helgoland. Prepared by ArcMap 10.4.1 (www.esri.de). Stacked bar plot represent 18S rDNA numbers of sequences and COI numbers of species per phylum at the stations sampled at autumn. Following abbreviations indicate stations: HTR, “Helgoländer Tiefe Rinne”; Loreley, Loreley Bank; HeN1 Helgoland North 01; HeN2, Helgoland North 02; HeW, Helgoland West. Prepared by Babett Günther using Microsoft Excel 2010 (www.microsoft.com).

In detail, this comparison is visualized for five samples from around Helgoland Island in autumn (Fig. 6). In all samples, 18S rDNA analyses detected Ctenophora, but COI analyses did not. Within the samples, 18S rDNA analyses detected Tunicata, but fewer or even no Craniata. In contrast, COI analyses only once detected tunicates (*Botryllus schlosseri*) but found three to six fish species, mostly *Pleuronectes platessa* and *Clupea harengus*, in all five samples. Station HTR had the lowest detection rates for arthropods in the 18S rDNA analyses and only seven species, whereas 14–25 species were detected at the other stations. The 25 species were found at the station Helgoland North 1, which is in line with the high relative abundance of arthropods according to the 18S rDNA analyses. No statistically significant correlation could be found between species composition and relative abundances of phyla, but a significant correlation (Mantel test, see Table 2) appeared between the 18S rDNA analyses and abiotic conditions (pressure, salinity, water density and temperature). Analyzing the abiotic factors single, the pressure, interpreted as sampling depth is the significant factor (Mantel test, p = 0.008).

**Table 2:** Mantel test results of similarity analyses for stations around Helgoland (see Fig. 6) for distances of stations and the combined as well as single abiotic factors measured by a CTD SONDE (compositional dissimilarity of pressure, temperature, salinity, water density).

	COI mDNA		18S rDNA	
	p-value	r	p-value	r
Abiotic factors	0.46	−0.14	0.01	0.67
Distances	0.13	0.75	0.79	−0.33
Pressure	0.46	−0.14	0.01	0.67
Salinity	0.04	0.71	0.94	−0.43
Water density	0.01	0.38	0.09	0.39
Temperature	0.42	−0.16	0.48	−0.03

Given are significances (p-value) and the correlation coefficient (r).

## Discussion

eDNA can be amplified and sequenced from quantities of seawater from a few milliliters to liters by means of modern high-throughput sequencing technologies for metagenomics analyses (see, e.g.,^15,18,19,21^; the study reported here). Barnes and Turner^8^ outlined the most important factors of the so called ecology of eDNA to be origin, state, transport, and the fate of the genetic material. The abundance and dispersion of eDNA depend of course on the DNA release and degradation in the water, but detection depends also on basic methodological issues like the size of the fragments and the filters systems used, as well as how and where the samples are taken and the transport of eDNA afterward. These factors could lead to wrong inferences about abundances and existence of organisms in our study. We began with equal samples of 1 L filtered water on the spring cruise. Especially for COI, few or no species could be recorded. For later samplings, we increased the filtered water to the maximum and used two filters per sample. The amount of filtrated water varied widely at different stations and seasons, depending on the concentration of phytoplankton, microorganisms, suspended particles, and solids in the seawater. Because it is so far impossible to work with absolute abundances, we saw no need to equalize the amount of sea water filtered; we simply adjusted the amount of DNA for analyzing samples from different locations and seasons. Mächler *et al*.^44^ analyzed the relationship between water volume sampled and detection rates. The number of sequences detected did not always increase with the volume of water, therefore the relation of species and their habitat is assumed to be an essential factor. For future studies, for stations with lower biomass, more water should be filtered, whereas for locations with high concentrations of suspended particles more than one filter should be used.

COI primer, the newly developed primer set for minibarcodes was highly efficient for the amplification of North Sea metazoans. The primers allowed the amplification of COI for diverse species from diverse phyla, and the 124-bp minibarcode fragment was used for the differentiation and identification of the different metazoan species. This primer set and fragment can therefore be used for future marine barcoding studies, both for single sequence analyses and for metabarcoding. However, it is known that using short length barcodes can reduce the efficiency of correct taxonomic identification^45,46^. Therefore, we recommend *in silico* analyses based on the reference database to ensure accurate taxonomic assignments.

18S primer, the nuclear 18S rDNA V8 fragment allowed amplification of a short fragment with a phylogenetic signal for all target phyla^29^, but as shown in the artificial sample, not every species could be detected or clearly identified. A metabarcoding study whose target was the natural zooplankton community from Helgoland used the hypervariable V1–V2 region with ~450 bp fragment^26^, but as in our study, not all species could be reliably differentiated, and not all species tested in the artificial sample could be confirmed. On the other hand, Guardiola *et al*.^24,25^ used the even shorter V7, only ~110 bp long, for metabarcoding eDNA of deepsea sediments and still produced reliable results for metazoans at the phylum level. Other regions, such as 12S rDNA^22^ and 16r DNA^23^ have also already proven to be feasible for eDNA in marine waters.

As a first step toward evaluating the biodiversity in environmental samples, we highly recommend the parallel analysis of artificial samples. Understanding the output of environmental sequences in terms of the relation of reads and OTUs is essential. On the basis of our artificial samples, we found no indication that the number of OTUs or sequences is correlated with amount of DNA and between gene regions. These results are in contrast to other studies, whose authors have assumed that abundances or biomass can be interpreted on the basis of the number of reads (e.g.,^15,17^). Hänfling *et al*.^16^, analyzing eDNA from a lake, found a significant correlation between the number of sequence reads and the amount of DNA in the template. Moreover the two mitochondrial gene regions (12S and cyt*b*) produced the same results. We attribute these differences from other studies to our use of a broad range of target metazoans and of a combination of nuclear and mitochondrial gene regions. Cowart *et al*.^47^ also found different frequencies of phyla when they compared results from COI, 18S rDNA, and morphological analysis. For the artificial samples, most of the obtained sequences could be identified (77% for 18S and 86% for COI, see Supplementary Information 4), indicating the efficiency of the metabarcoding approach. Nevertheless, for the environmental samples only 0 up to 2% (COI) or 20% (18S) of the sequences were identified. However, counted are only clear identified metazoan sequences. Based on the *in vitro* analyses both primers pairs were shown to be also efficient for other eukaryotic organisms, which were not in the focus of the study. The study of Stat *et al*.^48^ outlined how rare eukaryotic and especially metazoan eDNA is in marine water samples.

As mentioned above in the artificial sample of 18S rDNA amplifications, not all specimens could be detected. The necessary filtering of erroneous sequences (denoising) is known to cause loss of rare species^49^. On the other hand, a higher diversity of sequences and OUT’s are found than expected (see Supplementary Information 2). This probably by not detected sequencing errors, or chimeric sequences^50,51^. Using a similarity of 98% for assignment showed to exclude at least most of the wrong identifications. Exclusion of wrong positive detections can be further increased by inclusion of statistical model approaches^52^ or sequence error correction models^53,54^.

Analyzing extraorganismal eDNA from the 23 samples over three seasons in the German economic zone led to the final detection of 114 infaunal, pelagic and epibenthic species from a total of 12 phyla. Although, for marine eDNA water analyses, most studies analyzing 12S for species identification have focused on certain groups, such as fish or mammals^17,19,22^, O’Donnell *et al*.^23^ analyzed all metazoans but focused on spatial distribution of diversity, not on species level. The study reported here shows a COI-based short-length barcoding approach can identify a broad range of marine metazoan species with only one degenerated primer set.

Using the COI minibarcode primers we designed led to detection of 114 species, with a range of 0 to 60 detections per sample. A broad range from benthic to pelagic species was found, including 11 phyla, such as copepods, echinoderms, fish, and jellyfish. Infaunal phyla, like Polycheta, were also detected. Species were assigned with the help of two reference libraries, the COI EMBL reference database and the Senckenberg Barcode reference library of the North Sea. The latter database allows detection of more than 500 species, many more than comparable studies (e.g.,^17,19,22^). In addition, the North Sea metazoan database allows the precise assignment of species backed by expert knowledge and gives background information on the spatial distribution of species. On this basis, 80 species from seven phyla could be precisely assigned in 23 environmental samples from the 14 stations, which were part of the Long-term Ecological Research (LTER) network. The occurrence of species agreed (94.9%) with their occurrence at the LTER sites, and a large number of dominant species of the coastal benthic community in the southern North Sea were also found (e.g., *Ophiura ophiura*, *Asterias rubens*, *Astropecten irregularis*, *Buglossidium luteum*)^55^. Other species, such as the cephalopod *Sepiola tridens* detected during all seasons, especially around Helgoland, were not expected where we detected them. Studies have already reported that this species has been overlooked in the North Sea^40,56^ and is often wrongly identified as the morphologically similar *S*. *atlantica*, which we found only once But *S*. *tridens* has only been reported broadly for the northern part of the North Sea, not for the German exclusive economic zone we studied. Our results show that the distribution of this species extends much more widely into coastal areas than previously recorded.

Species assignment by means of the EMBL database allowed us to detect unexpected or probably invasive species in the North Sea, but especially in public sequence reference databases, sequences and the adequate assignment to species names must be handled with care. We have no *in silico* analyses for them, and we cannot confirm the power of species discrimination based on the chosen fragment. For example, the recorded bryozoan species *Tricellaria occidentalis* and *Crisia aculeata* are not known to occur in the North Sea, but their congeners *T*. *ternate* and *C*. *ebunea*^57^. Using the same similarity for assignment, this showing the advantage of the appropriate use of valid reference databases. We also recorded the crustacean *Artemia franciscana* in our German Bight samples. This American species is known to be invasive in hypersaline environments worldwide and has already been described from lakes along the Mediterranean coast^58^ of Spain and in the French part of the North Atlantic Ocean^59^. Although this species is unlikely to survive in the open ocean, it is among the most common crustaceans used as living food in aquaria worldwide. Already detected invasive species like the copepod *Oithona davisae*, previously recorded from the northern Wadden Sea at Sylt^60^, was detected in our data, as were the copepod *Pseudodiaptomus marinus*, also a new species recorded for the North Sea^61^, and the amphipod *Caprella mutica*, already described as neobiota in the North Sea^62^. Moreover, the cnidarian *Clytia languida* was identified. Species of *Clytia* are difficult to distinguish morphologically, so we cannot be certain whether this species is newly introduced to the North Sea or a sibling species of the common *C*. *hemisphaerica*, also detected^35,63^. Finally the combination of minibarcodes and the use of the Senckenberg Barcode reference library allowed species-level detection of metazoans and can also be applied to monitoring of invasive species in the North Sea, as it already is in fresh-water systems^64,65^.

In our data we observe differences in the number and frequency of molecular OTUs and thus species assignments detected by nuclear 18S V8 rDNA and mitochondrial COI analysis. These differences have already been observed in other metagenomic studies^47,66^. One reason is the affinity of the primers for the binding site, which is more conserved in nuclear than in mitochondrial genes. For example, the invasive ctenophore *Mnemiopsis leidyi* is known to occur in the North Sea^67^, was detected by our 18S rDNA analysis, and was highly abundant at this time around Helgoland (LTER, https://deims.org/1e96ef9b-0915-4661-849f-b3a72f5aa9b1). We did not, however, detect this species using minibarcode COI analyses. *In silico* tests using sequences from Genbank demonstrated that the minibarcode primers do not bind to this species’ COI sequence, explaining the difference in this species’ detection between these two gene fragments.

Nevertheless, combination of the two gene analyses also revealed similarities in the numbers of species and sequences detected, especially for arthropods and Cnidaria, as already outlined for samples around Helgoland. We benefit from the existence of comprehensive reference datasets for 18S and COI, especially in the cases of calanoid copepods^34^, the common jellyfish genus *Cyanea*^36^, and cephalopods^40^. Furthermore, both analyses giving different information but shown to be significant at seasonality. Reasons can be complex, by changes in biomass and community composition over the year. However, we suggest that this is mostly influenced by seasonal changes of the zooplankton community. As example, the 18S analyses of Fig. 4 visualize the change of higher Cnidarian detections in summer in contrast to more Ctenophora in the autumn.

The appliance of non-metric multidimensional scaling (nMDS, Fig. 5), shows the clustering of seasons but differences between the gene regions. As an example, the station Helgoland harbor (HH) in summer is the only sampling done directly within a harbor. The higher detection amount of tunicate is expected by the flat-water column with low current. Therefore, the 18S analyses show to be different from the others. But not for the species composition by COI. Here the station is more similar to the sampling site in front of the other sampled harbor (Jade). Therefore, similarity can be explained by the similar anthropogenic influence. Hence, the used gene regions can rather be understood as different perspectives on the sampling site. Eventually combing both gene regions can be used for further analyzing anthropogenic and climate impacts on the North Sea ecosystem. Like those of many other metabarcoding studies (see, e.g.^47^) our results confirm that eDNA monitoring surveys should use a multigene approach and be supported by comprehensive taxonomic reference libraries.

For future routine monitoring of marine communities, using extraorganismal eDNA is crucial to understanding the spatial resolution of the results^68^. Still unclear is whether the results show a snapshot of the contemporary species composition from the sampling site or represent a much wider spatial and temporal integration. In the North Sea, for example, the tidal current can lead to extensive drift and intense mixture of the “free“ DNA. Turner *et al*.^9^ have already described downward as well as horizontal eDNA transport. Moreover, they outlined the pitfall produced by resuspension of sedimentary eDNA into the water column, which can lead to wrong conclusions. Deiner & Altermatt^69^ reported that, in rivers, eDNA from invertebrates could be detected nearly 10 km from the source. eDNA can persist in aquatic systems for up to two weeks^8^ or even a month^70^. For marine systems, the reported persistence of eDNA is shorter (up to 7 days), and possible dispersion rates should be lower^15^. We therefore assume that eDNA detected in marine waters is more “local” than that in other aquatic systems and that the method may therefore have a more restricted detection area. On the basis of sampling from October around the island of Helgoland, where samples were taken at small spatial resolution, differences between the stations could still be detected, so we can assume local resolution (Fig. 6). In detail, the 18S rDNA results are significantly better correlated with abiotic factors than with the distances between the stations. The main factor was the pressure, indication that the depth of sampling and stations has a higher contribution than the distance. The 18S analyses as well as abiotic factors of the southern stations Helgoland west, and HTR are significant different compared to the northern stations. However, it is complex marine system and the results can only be reflecting the current around Helgoland. Detailed studies of the persistence and distribution of eDNA in the North Sea are required to evaluate the local resolution. eDNA will allow a potentially standardized biodiversity assessment and offer a tool with which to address many scientific questions like spatial distribution, community structure, habitat stability, and even impact of climate change or human influence.

## Conclusions

Our metagenomic study of extracellular eDNA allowed insights into the biodiversity of the North Sea metazoan fauna. Analyses of two gene regions display differences in species detection at different locations and different seasons. Analysis at the phylum level based on 18S rDNA V8 analyses could be compared to results produced with a COI primer developed for the purpose that allowed both the amplification of mitochondrial minibarcodes for diverse metazoan phyla and differentiation at the species level. In addition to species known to be distributed in this region, new benthic and pelagic species, some known from the North Sea and some not, were recorded with this molecular method. These applied methods offer a powerful tool for future biodiversity research including the detection of nonnative and invasive species. Nevertheless, a trustworthy species assignment depend on the validity and completeness of the sequence reference database.

## Methods

### Water sampling

Sampling in the German Bight was carried out on three cruises at different seasons (spring, summer, and autumn) of the RV *Senckenberg* in 2014, including 14 stations and 23 samples (see Supplementary Information 5, Fig. 2). Water samples were taken with a water sampler (1.7 L, General Oceanics, USA) 1 m above the bottom so that sediment would not be included in the samples. Samples were processed immediately on board and first prefiltered with sterile one-way plankton filters, first with a mesh size of 100 μm and then with a mesh size of 20 μm. Final filtering was performed with 0.1 μm sterile one way Rapid-Flow PES-membrane filters (Nalgene, USA) with a vacuum pump with at least 1 L (up to 1.7 L) of seawater from each station. In spring one filter was used per station, in summer and autumn two.

After filtration, filters were stored at −20 °C until extraction. Abiotic data were obtained in parallel for most stations with a CTD SONDE (CTD 48 M, Sea and Sun, Germany) an oceanographic instrument measure the pressure (indicating the depth), conductivity, salinity, temperature, voltage, and density of seawater (Supplementary Information 5).

### Extraction and pre-PCR

Each frozen filter was cut under sterile conditions under a lab hood into six parts, and each part was extracted separately with the NucleoSpin® Soil kit (Macherey Nagel, Germany) according to the manufacturer’s protocol. Using lysis buffer SL2 and the enhancer SX, we eluted DNA twice with 50 μl preheated molecular grade water. Blank samples (negative control) starting with the extraction done with molecular grade water were included and used for following PCR’s to exclude cross contaminations during the processing. All final extracts from one sample were pooled and reduced in volume by means of a vacuum centrifuge (30–45 °C). The DNA quantity of extracts and during the complete workflow up to library preparation was measured with a Qubit 3.0 Fluorometer (Invitrogen, Denmark) using the dsDNA BR Assay Kit (Invitrogen, Denmark). Fragment range was checked by gel electrophoresis in a 1–2.5% agarose gel with GelRed and commercial DNA size standards (0.1–2 kb).

In the marine environment, extracellular eDNA shows a high degree of damage, which can influence binding and amplification procedure of the polymerase^71,72^. Further DNA damage than fragmentation is expected, based on various abiotic and biotic degradation influences such as light and microorganisms^73^. To increase amplification success, we applied the PreCR Repair Mix (New England BioLabs, USA). This enzyme mix offers high-quality sequences^74^ by *in vitro* repair of abasic sites, nicks, thymidine dimers, blocked 3′-ends, oxidized guanine, oxidized pyrimidines, deaminated cytosine. According to the manufacturer’s protocol, each 50-µl PreCR reaction contained a maximum of 500 ng DNA per sample.

### Primers

COI. To detect a broad range of species with a focus on the macrofauna from the North Sea, we designed minibarcoding primers at the beginning of the study on the basis of the existing Senckenberg Barcode reference library, established for North Sea metazoans. That library included information on metadata, DNA sequences, DNA extracts, tissue samples, and voucher specimens. Most of these sequences are accessible on the Barcode of Life Data system (BOLD, http://www.boldsystems.org) under the following projects: Barcoding North Fish I (BNSFI), Barcoding North Sea Decapoda (BNAGB), Barcoding Gobiidae from the North Sea and the Baltic Sea (BGNBS), Barcoding Northeast Atlantic Cephalopoda (BNEAC), Barcoding Northeast Atlantic Gastropods and Bivalves (BNAGB), North Sea Echinodermata (DS-NSECH), Barcoding North Sea Cirripedia (BNSCI), Barcoding North Sea Isopoda (BNSIS), Barcoding North Sea Amphipoda (BNSA), Barcoding North Sea Copepoda (BNSCP), and Barcoding North Sea Crustacea (BNSC). The majority of the sequences are published, partially on NCBI^1,2,34–43^. The sequence reference library also includes some unpublished data on Cnidaria, Copepoda, and Polychaeta.

For *in silico* primer design, a final alignment including 3, 674 specimens from the North Sea was constructed with MUSCLE^75^, under use of the program Geneious^76^ version 7.0.4 (http://www.geneious.com). A suitable forward primer was found that began at position 196 bp in the 3′ direction and was named nsCOIFo (see Table 1). As a reverse primer, the universal metazoan primer mlCOIintR^31^ in the middle of the barcode fragment was used with a change of all five “W” positions to “S”, resulting in primer mlCOIintK (see Table 1). In order to verify species discrimination power of the short barcoding fragment (124 bp), we performed *in silico* primer tests with a species consensus (100% threshold for sequences) alignment (n = 506).

18S For the 18S analyses, we used the primers 18S #4 and 18S #5_RC for amplification of the V8 region developed by Machida & Knowlton^29^ (Table 1).

### Tagging and pooling

All primers were tagged (marked with MID, Multiplex Identifier) five times at the 5′ end (Table 1), for pooling of samples before library preparation. This index set up allow the pooling directly after PCR and reduce the contamination risk. For COI primers, 6-bp tags were created that had no more than two identical bases. The tagging for 18S was reduced to 4 bp because of the longer fragment. We took care that the contents of purines and pyrimidines were equal, without direct repetitions of bases. Primers were produced by Biomers (Germany). The concentration of final prepared primer mixes was 20 ng/µl.

### Test of primers

To test the efficiency of primers *in vitro* and to optimize PCR settings, we used a set of 30 DNA extracts including a broad range of North Sea metazoan taxa like cnidarians, fishes, mollusks, crustaceans, and echinoderms (Supplementary Information 1) potentially present in the water samples. We also tested 15 nontarget DNA extracts, including human, algae, bacteria, and dinoflagellates from the North Sea. The extraction of the nontargets was performed with the NucleoSpin® Soil kit (Macherey Nagel, Germany).

PCRs included 15 pmol of each primer, 15 µl AccuStart II PCR ToughMix (Quantabio, USA), 2–4 µl DNA extract, and sufficient molecular water to yield a final volume of 25 µl. PCR cycling conditions were 94 °C for 3 min followed by 35 cycles of 94 °C for 30 s, 43 °C for 30 s, 72 °C for 30 s, and a final elongation at 72 °C for 5 min. All PCR’s included positive and negative controls. The PCR products were purified with ExoSAP-It (AppliChem, Germany) and sequenced by Sanger technology (Macrogen, Netherlands).

### Environmental samples for Illumina sequencing

For high-throughput sequencing we prepared the 23 (see Supplementary Information 5) environmental samples and one artificial community as *in situ* test with an equalized mixture of the 30 target DNA extracts (Supplementary Information 1). The latter contained 5 ng DNA per extract, yielding a final concentration of 0.9 ng/µl. Every sample was amplified with COI as well as 18S primers. For minimum PCR bias and detection of the majority of species within one sample, a minimum of eight replicates is recommended^77^. We performed 12 replicates for every PCR, in order to amplify DNA from as many species as possible.

Each PCR reaction included 1 µl of the PreCR (~10 ng/µl DNA) treated DNA template, 15 pmol primer mix, 7.5 µl AccuStart II PCR ToughMix, and sufficient molecular water to yield a final volume of 15 µl. To ensure amplification, we increased the amount of DNA for the artificial sample to 6 µl. PCR cycling conditions were 94 °C for 3 min followed by 25 cycles of 94 °C for 30 s, 43 °C for 30 s, 72 °C for 30 s, and a final elongation at 72 °C for 5 min. The number of cycles had been previously optimized by means of tests running from 5 to 30 cycles, by gel electrophoresis and Qubit measurements. The idea is to avoid the last steps of massive replication at a PCR and to increase the diversity of sequences. All PCR’s included negative controls.

All 12 PCR products from each sample were merged and loaded on a 2.5% agarose gel with concentrated loading dye for size selection. We cut out the target fragment range and extracted it with the NucleoSpin Gel and PCR Clean-up kit (Macherey Nagel, Germany), by applying the wash steps and elution in 30 µl molecular water twice. The final volume was reduced at 45 °C in a vacuum centrifuge.

The amount of DNA required for a sequencing-library preparation was 500 ng. From each of the 24 samples, 30 ng of the products were pooled to form one sample for each of the two gene regions. The final concentration of both pooled samples was increased to more than 10 ng/µl by vacuum centrifugation at 45 °C. Library preparation and sequencing were performed by GATC BIOTECH (Germany). Samples were sequenced with an Illumina Hiseq2500, using the 125-bp paired-end reading and a minimum of 10 M read pairs per library. Within libraries, 20% of PhiX control was included, providing a quality control for Illumina sequencing and to increase library diversity.

### Quality check of sequences

Sequences produced with Sanger technology were assembled and checked manually with Geneious. For handling Illumina output, we used the program pipeline OBITools (metabarcoding.org/obitools/doc/index.html) and corresponding available scripts^78,79^. The assembly of the forward and reverse reads was performed with the program illuminapairedend by application of a minimum quality score of 30, allowing up to four mismatches in primer sequences, but no mismatch within the tags. Unaligned sequences were filtered out with obigrep. The data were then demultiplexed into the original samples with the program obisplit. The quality filter required sequence length of 100 bp for COI, 140 bp for 18S, and a minimum of three reads. All identical sequences (100%) in one sample were clustered together and handled thereafter as operational taxonomic units (OTUs). Identical sequences of different lengths were counted as different OTUs. For exclusion of amplification and sequencing errors (denoising), a directed acyclic graph based on amount and similarity of sequences was created by obiclean, using a treshold of r = 0.05. Every OTU was labeled as “head,” (frequent sequence) “singleton”, (sequence lacking a variant) or “internal”^78^. All internal OTUs were discarded, as most probably corresponded to PCR artifacts like chimeras.

### Reference data for taxonomic assignment

For the taxonomic assignment of environmental samples, we used the Senckenberg Barcode reference library from the North Sea, which was already used for the COI primer design. In addition, for each marker system a reference library based on standard European Molecular Biology Laboratory (EMBL) sequences (September 2016, European Nucleotide Archive, ENA Release 128) was created. Virtual PCR was performed allowing four mismatches per primer with *ecoPCR*^80,81^. National Center for Biotechnology Information taxonomy was included to assign sequence/specimen to their full taxonomic lineage. The final EMBL reference databases for COI as well as 18S included only metazoan sequences and all potential fragments based on the primers used.

To check the efficiency of the primer pairs in the artificial samples *in situ*, we used the known sequences of the target specimens as a reference database. They were based on the Senckenberg Barcode reference library as well as the sequences of the test PCRs.

### Taxonomic assignment and evaluation of the data

The assignments of the artificial samples (COI and 18S) and the environmental COI detections against the Senckenberg Barcode reference library was performed by the cluster program cd-hit-est-2d^82^. To check against the EMBL reference databases, we used the program *ecotag*^78^. All assignments were performed with a 98% similarity rate. Somes samples were shown human detections in low OTU amounts. However, general contamination could be excluded as all blank samples and subsequent negative conrtols shown no contamination at all. For all taxonomic assignments, detection of *Homo sapiens* was always deleted.

On the basis of the taxonomic lineages, every OTU was added to one of the following higher taxonomic groups (based on the tree of Metazoa from the National Center for Biotechnology Information): Annelida, Arthropoda, Bryozoa, Chaetognatha, Cnidaria, Craniata, Ctenophora, Echinodermata, Entoprocta, Gastrotricha, Hemichordata, Mollusca, Nematoda, Nemertea, Platyhelminthes, Porifera, Rotifera, Tunicata, Xenacoelomorpha. OTUs that could not be assigned to one of these groups were deleted from further analyses. They are mainly identified and suggested as prokaryotic and other detections based on the positive results of the non-target tests, and out of the focus of this study. The amount of detected sequences and OUTs after bioinformatical processing and final identified per sample is given in Supplementary Information 4. Applied for 18S was an additional deletion of taxon groups with less than 2% abundance of reads in a sample^50^ so as to exclude presence of artifactual and false-positive taxa^51,83^. This enable to focus on the primary abundant phyla for analysis. For COI, this was not necessary as identified species could be verified by the LTER.

### Statistical analyses

For statistical analyses, we used R-Studio (Version 0.98.1103), including the packages MASS and vegan^84,85^. The saturation of metazoan detection checked via rarefaction for the EMBL based taxonomic assignments. Therefore, OTU tables were combined for every gene region separately, based on the taxa identification number of NCBI. Rarefaction curves prepared with ggplot2^86^ showing the amount of detected taxa to the amount of identified sequences per sample (Supplementary Information 6).

For 18S rDNA data, the number of sequences, as well as abiotic measurements of the CTD SONDE, were standardized (equalized unit total). Euclidean distance, which quantifies the compositional dissimilarity, was used to analyze the pattern of phyla among the sampling sites and to create a distance matrix of the stations. For COI presence-absence comparison, the Sørensen index was applied^87^. These analyses were compared by Mantel test for distinct stations around the island Helgoland.

Analyses of seasonality were carried out with PRIMER v6 software with the PERMANOVA+ add-on package^88,89^. The homogeneity of sample dispersions was tested by distance-based PERMDISP tests (distances to centroid). We analyzed the differences among communities and conducted pair-wise tests between seasons by multivariate nonparametric permutational ANOVA (PERMANOVA with 9999 permutations). Combining the two tests allowed us to determine whether the groups of seasons differed in their location, in their dispersion, or in a combination of the two^89^. The seasonal analyses were visualized by non-metric multidimensional scaling (nMDS)^90^ analysis using the mentioned Sørensen index for COI and Euclidean distance for 18S^52^.

## Electronic supplementary material

Supplementary Information

## Data Availability

Raw Illumina sequences are available in NCBI SRA (Sequence Read Archive, Accession: PRJNA485040). The used Senckenberg Barcode reference library is available as fasta file by request at the corresponding author.
