# Publisher Correction: Metabarcoding of marine environmental DNA based on mitochondrial and nuclear genes

**DOI:** 10.1038/s41598-019-46784-7

**Published:** 2019-07-18

**Authors:** Babett Günther, Thomas Knebelsberger, Hermann Neumann, Silke Laakmann, Pedro Martínez Arbizu

**Affiliations:** 1 Senckenberg am Meer, German Centre for Marine Biodiversity Research, Südstrand 44, 26382 Wilhelmshaven, Germany; 2 Present Address: Ifremer (Institut français de recherche pour l’exploitation de la mer), Avenue Jean Monnet BP 171, 34200 Sète, France; 3 Senckenberg am Meer, Marine Research, Südstrand 40, 26382 Wilhelmshaven, Germany; 4 Present Address: Helmholtz Institute for Functional Marine Biodiversity at the University of Oldenburg (HIFMB), Ammerländer Heerstraße 231, 26129 Oldenburg, Germany

Correction to: *Scientific Reports* 10.1038/s41598-018-32917-x, published online 04 October 2018

The original version of this Article contained an error in Affiliation 2 which was incorrectly given as ‘Ifremer (Institut français de recherche pour l’exploitation de la mer), Avenue Jean Monnet, 34200, Sète, France’. The correct affiliation is listed below:

Ifremer (Institut français de recherche pour l’exploitation de la mer), Avenue Jean Monnet BP 171, 34200, Sète, France.

Additionally, in the original version of this Article, Hermann Neumann was incorrectly affiliated with ‘Ifremer (Institut français de recherche pour l’exploitation de la mer), Avenue Jean Monnet, 34200, Sète, France’.

Furthermore, in the original HTML version of this Article, an affiliation was omitted for Babett Günther. The correct affiliations for Babett Günther are listed below:

Senckenberg am Meer, German Centre for Marine Biodiversity Research, Südstrand 44, 26382, Wilhelmshaven, Germany.

Present address: Ifremer (Institut français de recherche pour l’exploitation de la mer), Avenue Jean Monnet BP 171, 34200, Sète, France.

These errors have now been corrected in the PDF and HTML versions of the Article.

