# Supplementary material for: Metabarcoding of marine environmental DNA based on mitochondrial and nuclear genes
Source: Sci Rep. 2018 Oct 4;8:14822. doi: 10.1038/s41598-018-32917-x (PMC6172225; doi:10.1038/s41598-018-32917-x)

**Supplementary Information**

**Manuscript:** Metabarcoding of marine environmental DNA based on mitochondrial and nuclear genes

**Authors:** Babett Günther, Thomas Knebelsberger , Hermann Neumann , Silke Laakmann , Pedro Martínez Arbizu

**Supplementary Information 1.**

Output of the *in vitro* tests with primer pairs for target and nontarget DNA extracts. Symbols: +, postive amplification; -, negative amplification; *; positive but differ in fragment sizes; /, no primer mismatch at all

|  |  | **Group** | **Species/Genus** | **Sample no.** | **Source** | **Amplifikation sucsess** | | **Missmatches in silico** | |
| --- | --- | --- | --- | --- | --- | --- | --- | --- | --- |
|  |  |  |  |  |  | #F4/ #F5_RC | mlCOIintK/  nsCOIFo | nsCOIFo | mlCOIintK |
| **Non target** | Dinoflagellata | Dinophyceae | *Tripus horridus* | - | Marine Botany,Senckenberg am Meer, German Centre for Marine Biodiversity Research | + | - |  |  |
|  |  |  | *Heterocapsa triquetra* | - |  | + | * |  |  |
|  |  |  | *Prorocentrum micans* | - |  | + | + |  |  |
|  |  |  | *Scrippsiella acuminata* | - |  | + | - |  |  |
|  | Bacteria | Alphaproteobacteria | *Leisingera nanhaiensis* | - | Biology of Geological Processes - Aquatic Microbial Ecology, The Institute for Chemistry and Biology of the Marine Environment (ICBM) | * | + |  |  |
|  |  | Alphaproteobacteria | *Pheaobacter gallaeciensis* | - |  | * | * |  |  |
|  |  | Gammaproteobacteria | *Vibrio coralliytrans* | - |  | + | - |  |  |
|  |  | *Flavobacterium* | *Winogradskyella ulvae* | - |  | + | * |  |  |
|  | Algae | Rotifera | *Brachionus*  sp. | - | Plankton Ecology Lab, The Institute for Chemistry and Biology of the Marine Environment (ICBM) | + | + |  |  |
|  |  | Diatomeen/ Bacillariophyta | *Chaetoceros* sp. | - |  | + | + |  |  |
|  |  |  | *Cylindrotheca closterium* | - |  | + | + |  |  |
|  |  | Raphidophyceae | *Fibrocapsa japonica* | - |  | + | + |  |  |
|  |  |  | *Rhodomonas salina* | - |  | + | + |  |  |
|  |  | Cyanobacteria | *Synechococcus* sp. | - |  | * | * |  |  |
|  | Human | Mammalia | *Homo sapiens* | - | intern | + | + |  |  |
| **Target** | Metazoa | Actinoterygii | *Clupea harengus* | MT01760 | BNSF308-11 | + | + | 1 | 4 |
|  |  | Actinoterygii | *Pleuronectes platessa* | MT01305 | BNSF061-11 | + | + | - | 2 |
|  |  | Amphipoda | *Caprella mutica* | MT01237 | BNSC162-10 | + | + | - | 3 |
|  |  | Amphipoda | *Jassa herdmani* | MT02760 | BNSA100-12 | + | + | 1 | 1 |
|  |  | bivalvia | *Venus casina* | MT04847 | BNAGB181-14 | + | + | - | 3 |
|  |  | bivalvia | *Crassostrea gigas* | MT09440 | BNAGB696-14 | + | + | - | 4 |
|  |  | Cephalopoden | *Sepiola tridens* | MT03073 | BNEAC037-13 | + | + | - | - |
|  |  | Cephalopoden | *Loligo forbesii* | MT04918 | BNEAC066-13 | + | + | - | - |
|  |  | Copepoda | *Temora longicornis* | MT00499 | BNSCP034-11 | + | + | 1 | 2 |
|  |  | Copepoda | *Lernaeenicus sprattae* | MT08939 | BNSCP115-14 | + | + | 1 | 2 |
|  |  | Cumacea | *Bodotria scorpioides* | MT01573 | BNSC312-11 | + | + | - | 4 |
|  |  | Decapoda | *Liocarcinus depurator* | MT01292 | BNSC270-11 | + | + | - | 1 |
|  |  | Decapoda | *Cancer pagurus* | MT03197 | BNSDE239-12 | + | + | - | 2 |
|  |  | Diplostraca | *Evadne spinifera* | MT03907 | BNSC366-11 | + | + | - | 4 |
|  |  | Dipostraca | *Podon leuckartii* | MT01579 | BNSC293-11 | + | + | - | 2 |
|  |  | Echinodermata | *Ophiura ophiura* | MT03600 | Unpublic, intern | + | + | - | 1 |
|  |  | Echinodermata | *Asterias rubens* | MT03741 | NSECH031-13 | + | + | - | 3 |
|  |  | Echinodermata | *Echinocyamus pusillus* | MT02275 | BNSE059-11 | + | + | - | 3 |
|  |  | Elasmobranchii | *Raja clavata* | MT02869 | BNSF449-12 | + | + | - | 3 |
|  |  | Gastrophoda | *Littorina littorea* | MT09395 | BNAGB651-14 | + | + | - | 2 |
|  |  | Gastrophoda | *Doto coronate* | MT09696 | BNAGB872-14 | + | + | - | - |
|  |  | Hydrozoa | *Aurelia aurita* | MT03817 | Unpublic, intern | + | + | 1 | 2 |
|  |  | Hydrozoa | *Aglantha digitale* | MT06853 | Unpublic, intern | + | + | 3 | 5 |
|  |  | Isopoda | *Idotea baltica* | MT01194 | BNSC119-10 | + | + | - | 1 |
|  |  | Mysida | *Mesopodopsis slabberi* | MT02064 | BNSC395-12 | + | + | 1 | 3 |
|  |  | Mysida | *Praunus flexuosus* | MT08547 | BNSDE373-14 | + | + | - | 3 |
|  |  | Oligochaeta | *Clitellio arenarius* | MT03448 | BNSP051-11 | + | + | 2 | 1 |
|  |  | Polycheta | *Ophelina acuminata* | MT03439 | Unpublic, intern | + | + | 2 | 3 |
|  |  | Polycheta | *Tomopteris cf. Helgolandica* | MT03457 | Unpublic, intern | + | + | - | - |
|  |  | Thecostraca | *Balanus crenatus* | MT04577 | BNSC461-12 | + | + | - | - |

.

**Supplementary Information 2**


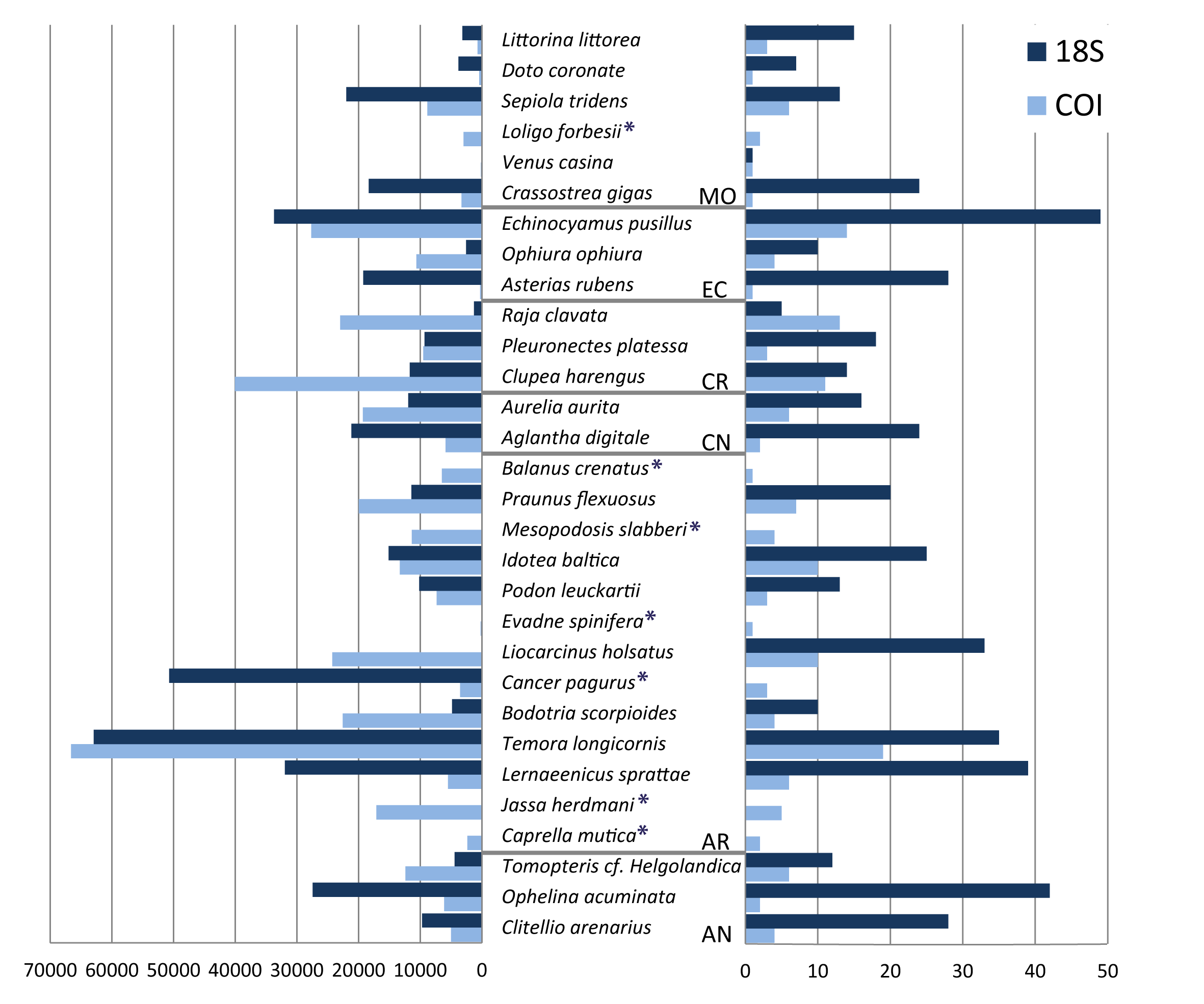


Results of Illumina sequencing of target specimens in the artificial sample. The left side shows the final number of reads per specimen. The right side shows the number of operational taxonomic units per specimen. For COI all specimens could be detected in case of 18S analyses not identified specimens are labeled with asterisks (*). Boldface letters indicate higher taxa: MO, Mollusca; EC, Echinodermata; CR, Craniata; CN, Cnidaria; AR, Arthropoda; AN, Annelida.

**Supplementary Information 3.** List of species detected within the environmental samples by means of minilength barcodes. Analysis based on the taxonomic assignment with the North Sea database, additional identified species with EMBL database are shaded in grey. The letters besides species indicate higher taxa: AN, Annelida; AR, Arthropoda; BR, Bryozoa; Ch, Chaetognatha; CN, Cnidaria; CR, Craniata; EC, Echinodermata; MO, Mollusca; Ne, Nemertea; Po, Porifera; Tu, Tunicata.

Spring

| **Station** | **Station 37** |  | **Station 23** |  | **BOX A** |  | **Elbe** |  |
| --- | --- | --- | --- | --- | --- | --- | --- | --- |
| **Sample No.** | **1** |  | **2** |  | **3** |  | **5** |  |
|  | *Oithona similis* | Ar | *Lanice conchilega* | An | *Lagis koreni* | An | *Magelona mirabilis** | An |
|  | *Paracalanus parvus* | Ar | *Oithona similis* | Ar | *Lanice conchilega* | An | *Tomopteris cf. Helgolandica* | An |
|  | *Echinocardium cordatum* | Ec | *Paracalanus parvus* | Ar | *Malacoceros cf. tetracerus* | An | *Balanus crenatus* | Ar |
|  |  |  | *Aglantha digitale* | Cn | *Calanus helgolandicus* | Ar | *Bodotria scorpioides* | Ar |
|  |  |  | *Limanda limanda* | Cr | *Evadne nordmanni* | Ar | *Idotea balthica* | Ar |
|  |  |  | *Microstomus kitt* | Cr | *Paracalanus parvus* | Ar | *Jassa herdmani* | Ar |
|  |  |  | *Pleuronectes platessa* | Cr | *Pseudocalanus elongatus* | Ar | *Liocarcinus depurator* | Ar |
|  |  |  | *Echinocardium cordatum* | Ec | *Temora longicornis* | Ar | *Praunus flexuosus* | Ar |
|  |  |  | *Ophiothrix fragilis* | Ec | *Echinocardium cordatum* | Ec | *Temora longicornis* | Ar |
|  |  |  | *Temora longicornis* | Ar | *Ophiura ophiura* | Ec | *Aurelia aurita* | Cn |
|  |  |  |  |  | *Lutraria lutraria* | Mo | *Clupea harengus* | Cr |
|  |  |  |  |  | *Pectinaria koreni* | An | *Pleuronectes platessa* | Cr |
|  |  |  |  |  | *Spiophanes bombyx* | An | *Raja clavata* | Cr |
|  |  |  |  |  |  |  | *Echinocyamus pusillus* | Ec |
|  |  |  |  |  |  |  | *Ophiura ophiura* | Ec |
|  |  |  |  |  |  |  | *Sepiola tridens*⁰ | Mo |
|  |  |  |  |  |  |  | *Magelona johnstoni* | An |
|  |  |  |  |  |  |  | *Ophiura albida* | Ec |

Summer

| **Station 37** |  | **Station 23** |  | **BOX A** |  | **HTR** |  | **Jade** |  | **Station 34** |  | **Station 16** |  | **Loreley Bank** |  | **Helgoland Hafen** |  |
| --- | --- | --- | --- | --- | --- | --- | --- | --- | --- | --- | --- | --- | --- | --- | --- | --- | --- |
| **7** |  | **9** |  | **10** |  | **11** |  | **12** |  | **13** |  | **14** |  | **15** |  | **16** |  |
| *Echinocardium cordatum* | Ec | *Aglantha digitale* | Cn | *Tomopteris cf. Helgolandica* | An | *Acartia clausi* | Ar | *Balanus balanus* | Ar | *Oithona similis* | Ar | *Paracalanus parvus* | Ar | *Clitellio arenarius*⁰ | An | *Aurelia aurita* | Cn |
|  |  | *Aurelia aurita* | Cn | *Balanus balanus* | Ar | *Aurelia aurita* | Cn | *Aglantha digitale* | Cn | *Paracalanus parvus* | Ar | *Pseudocalanus elongatus* | Ar | *Ophelina acuminata* | An | *Eucheilota maculata* | Cn |
|  |  | *Cyanea capillata* | Cn | *Bodotria scorpioides* | Ar | *Clytia hemispherica* | Cn | *Eucheilota maculata* | Cn | *Aglantha digitale* | Cn | *Aurelia aurita* | Cn | *Tomopteris cf. Helgolandica* | An | *Ascidiella aspersa* | Tu |
|  |  | *Melicertum octocostatum* | Cn | *Idotea balthica* | Ar | *Eucheilota maculata* | Cn | *Eutima gegenbauri* | Cn | *Cyanea capillata* | Cn | *Eutima gracilis* | Cn | *Balanus balanus* | Ar | *Pomatoceros triqueter* | An |
|  |  | *Hubrechtella dubia⁰* | Ne | *Jassa herdmani* | Ar | *Eutima gegenbauri* | Cn | *Leuckartiara octona* | Cn | *Eucheilota maculata* | Cn | *Helgicirrha schulzei* | Cn | *Balanus crenatus* | Ar | *Sagartia ornata* ^2^ | Cn |
|  |  |  |  | *Paracalanus parvus* | Ar | *Helgicirrha schulzei* | Cn | *Nemopsis bachei* | Cn | *Leuckartiara octona* | Cn | *Limanda limanda* | Cr | *Bodotria scorpioides* | Ar |  |  |
|  |  |  |  | *Podon leuckartii* | Ar | *Leuckartiara octona* | Cn |  |  | *Arnoglossus laterna* | Cr | *Tenuilineus albocinctus*⁰ | Ne | *Cancer pagurus* | Ar |  |  |
|  |  |  |  | *Temora longicornis* | Ar | *Lizzia blondina* | Cn |  |  | *Buglossidium luteum* | Cr |  |  | *Caprella mutica* | Ar |  |  |
|  |  |  |  | *Aurelia aurita* | Cn | *Obelia lanaissima* | Cn |  |  | *Microstomus kitt* | Cr |  |  | *Idotea balthica* | Ar |  |  |
|  |  |  |  | *Eutima gracilis* | Cn | *Limanda limanda* | Cr |  |  | *Echinocardium cordatum* | Ec |  |  | *Jassa herdmani* | Ar |  |  |
|  |  |  |  | *Helgicirrha schulzei* | Cn | *Pleuronectes platessa* | Cr |  |  | *Spirobranchus triqueter* | An |  |  | *Lernaeenicus sprattae⁰* | Ar |  |  |
|  |  |  |  | *Leuckartiara octona* | Cn | *Abra alba* | Mo |  |  |  |  |  |  | *Liocarcinus depurator* | Ar |  |  |
|  |  |  |  | *Buglossidium luteum* | Cr | *Abra nitida* | Mo |  |  |  |  |  |  | *Mesopodopsis slabberi* | Ar |  |  |
|  |  |  |  | *Clupea harengus* | Cr | *Sagartia ornata* ^2^ | Cn |  |  |  |  |  |  | *Podon leuckartii* | Ar |  |  |
|  |  |  |  | *Limanda limanda* | Cr |  |  |  |  |  |  |  |  | *Praunus flexuosus* | Ar |  |  |
|  |  |  |  | *Pleuronectes platessa* | Cr |  |  |  |  |  |  |  |  | *Pseudocalanus elongatus* | Ar |  |  |
|  |  |  |  | *Echinocyamus pusillus* | Ec |  |  |  |  |  |  |  |  | *Temora longicornis* | Ar |  |  |
|  |  |  |  | *Sepiola tridens⁰* | Mo |  |  |  |  |  |  |  |  | *Aglantha digitale* | Cn |  |  |
|  |  |  |  | *Electra sp. LM-2010* ^1^ | Br |  |  |  |  |  |  |  |  | *Aurelia aurita* | Cn |  |  |
|  |  |  |  | *Sagitta setosa* | Ch |  |  |  |  |  |  |  |  | *Eucheilota maculata* | Cn |  |  |
|  |  |  |  | *Botryllus schlosseri* | Tu |  |  |  |  |  |  |  |  | *Eutima gegenbauri* | Cn |  |  |
|  |  |  |  |  |  |  |  |  |  |  |  |  |  | *Leuckartiara octona* | Cn |  |  |
|  |  |  |  |  |  |  |  |  |  |  |  |  |  | *Buglossidium luteum* | Cr |  |  |
|  |  |  |  |  |  |  |  |  |  |  |  |  |  | *Clupea harengus* | Cr |  |  |
|  |  |  |  |  |  |  |  |  |  |  |  |  |  | *Limanda limanda* | Cr |  |  |
|  |  |  |  |  |  |  |  |  |  |  |  |  |  | *Pleuronectes platessa* | Cr |  |  |
|  |  |  |  |  |  |  |  |  |  |  |  |  |  | *Pomatoschistus minutus* | Cr |  |  |
|  |  |  |  |  |  |  |  |  |  |  |  |  |  | *Raja clavata* | Cr |  |  |
|  |  |  |  |  |  |  |  |  |  |  |  |  |  | *Astropecten irregularis* | Ec |  |  |
|  |  |  |  |  |  |  |  |  |  |  |  |  |  | *Echinocardium cordatum* | Ec |  |  |
|  |  |  |  |  |  |  |  |  |  |  |  |  |  | *Echinocyamus pusillus* | Ec |  |  |
|  |  |  |  |  |  |  |  |  |  |  |  |  |  | *Ophiura ophiura* | Ec |  |  |
|  |  |  |  |  |  |  |  |  |  |  |  |  |  | *Crassostrea gigas* | Mo |  |  |
|  |  |  |  |  |  |  |  |  |  |  |  |  |  | *Loligo forbesii* | Mo |  |  |
|  |  |  |  |  |  |  |  |  |  |  |  |  |  | *Sepiola tridens*⁰ | Mo |  |  |
|  |  |  |  |  |  |  |  |  |  |  |  |  |  | *Electra sp. LM-2010* ^1^ | Br |  |  |
|  |  |  |  |  |  |  |  |  |  |  |  |  |  | *Sagartia ornata* ^2^ | Cn |  |  |
|  |  |  |  |  |  |  |  |  |  |  |  |  |  | *Sagitta setosa* | Ch |  |  |
|  |  |  |  |  |  |  |  |  |  |  |  |  |  | *Tenuilineus albocinctus*⁰ | Ne |  |  |

Autumn

| **BOX A** |  | **HTR** |  | **Jade** |  | **Loreley Bank** |  | **Helgoland North 01** |  | **Helgoland North 02** |  | **Helgoland West** |  |
| --- | --- | --- | --- | --- | --- | --- | --- | --- | --- | --- | --- | --- | --- |
| **17** |  | **18** |  | **19** |  | **20** |  | **21** |  | **22** |  | **23** |  |
| *Balanus balanus* | Ar | *Lanice conchilega* | An | *Arenicola marina* | An | *Clitellio arenarius*⁰ | An | *Clitellio arenarius*⁰ | An | *Clitellio arenarius*⁰ | An | *Clitellio arenarius*⁰ | *An* |
| *Paracalanus parvus* | Ar | *Amphibalanus improvisus* | Ar | *Balanus balanus* | Ar | *Ophelina acuminata* | An | *Ophelina acuminata* | An | *Ophelina acuminata* | An | *Ophelina acuminata* | *An* |
| *Penilia avirostris* | Ar | *Aora gracilis* | Ar | *Aurelia aurita* | Cn | *Tomopteris cf. Helgolandica* | An | *Scalibregma inflatum* | An | *Scalibregma inflatum* | An | *Scalibregma inflatum* | *An* |
| *Clytia hemispherica* | Cn | *Balanus balanus* | Ar | *Tricellaria occidentalis*³ | Br | *Balanus balanus* | Ar | *Tomopteris cf. Helgolandica* | An | *Tomopteris cf. Helgolandica* | An | *Tomopteris cf. Helgolandica* | *An* |
| *Clytia languida* | Cn | *Elminius modestus* | Ar |  |  | *Balanus crenatus* | Ar | *Amphibalanus improvisus* | Ar | *Amphibalanus improvisus* | Ar | *Balanus balanus* | *Ar* |
| *Eucheilota maculata* | Cn | *Paracalanus parvus* | Ar |  |  | *Bodotria scorpioides* | Ar | *Aora gracilis* | Ar | *Balanus balanus* | Ar | *Balanus crenatus* | *Ar* |
| *Nucula nitidosa* | Mo | *Penilia avirostris* | Ar |  |  | *Cancer pagurus* | Ar | *Balanus balanus* | Ar | *Balanus crenatus* | Ar | *Bodotria scorpioides* | *Ar* |
| *Protodrilus oculifer*⁰ | An | *Temora longicornis* | Ar |  |  | *Caprella mutica* | Ar | *Balanus crenatus* | Ar | *Bodotria scorpioides* | Ar | *Cancer pagurus* | *Ar* |
| *Electra sp. LM-2010* ^1^ | Br | *Aglantha digitale* | Cn |  |  | *Elminius modestus* | Ar | *Bodotria scorpioides* | Ar | *Cancer pagurus* | Ar | *Caprella mutica* | *Ar* |
| *Tricellaria occidentalis*³ | Br | *Eucheilota maculata* | Cn |  |  | *Idotea balthica* | Ar | *Cancer pagurus* | Ar | *Caprella mutica* | Ar | *Idotea balthica* | *Ar* |
| *Sagitta setosa* | Ch | *Eutima gegenbauri* | Cn |  |  | *Jassa herdmani* | Ar | *Caprella mutica* | Ar | *Crangon allmanni* | Ar | *Jassa herdmani* | *Ar* |
| *Pelagia noctiluca* ⁰ | Cn | *Helgicirrha schulzei* | Cn |  |  | *Lernaeenicus sprattae*⁰ | Ar | *Crangon allmanni* | Ar | *Elminius modestus* | Ar | *Lernaeenicus sprattae*⁰ | *Ar* |
|  |  | *Leuckartiara octona* | Cn |  |  | *Liocarcinus depurator* | Ar | *Elminius modestus* | Ar | *Idotea balthica* | Ar | *Liocarcinus depurator* | *Ar* |
|  |  | *Obelia dichotoma* | Cn |  |  | *Mesopodopsis slabberi* | Ar | *Euterpina acutifrons* | Ar | *Jassa herdmani* | Ar | *Mesopodopsis slabberi* | *Ar* |
|  |  | *Agonus cataphractus* | Cr |  |  | *Podon leuckartii* | Ar | *Idotea balthica* | Ar | *Lernaeenicus sprattae⁰* | Ar | *Paracalanus parvus* | *Ar* |
|  |  | *Buglossidium luteum* | Cr |  |  | *Praunus flexuosus* | Ar | *Jassa herdmani* | Ar | *Liocarcinus depurator* | Ar | *Podon leuckartii* | *Ar* |
|  |  | *Callionymus lyra* | Cr |  |  | *Temora longicornis* | Ar | *Lernaeenicus sprattae*⁰ | Ar | *Mesopodopsis slabberi* | Ar | *Praunus flexuosus* | *Ar* |
|  |  | *Limanda limanda* | Cr |  |  | *Aglantha digitale* | Cn | *Liocarcinus depurator* | Ar | *Pandalus montagui* | Ar | *Temora longicornis* | *Ar* |
|  |  | *Pleuronectes platessa* | Cr |  |  | *Aurelia aurita* | Cn | *Mesopodopsis slabberi* | Ar | *Penilia avirostris* | Ar | *Aglantha digitale* | *Cn* |
|  |  | *Astropecten irregularis* | Ec |  |  | *Clytia hemispherica* | Cn | *Monocorophium sextonae* | Ar | *Podon leuckartii* | Ar | *Aurelia aurita* | *Cn* |
|  |  | *Phaxas pellucidus* | Mo |  |  | *Eucheilota maculata* | Cn | *Pandalus montagui* | Ar | *Praunus flexuosus* | Ar | *Clytia hemispherica* | *Cn* |
|  |  | *Sepiola atlantica* | Mo |  |  | *Leuckartiara octona* | Cn | *Penilia avirostris* | Ar | *Temora longicornis* | Ar | *Eucheilota maculata* | *Cn* |
|  |  | *Pomatoceros triqueter* | An |  |  | *Clupea harengus* | Cr | *Podon leuckartii* | Ar | *Aglantha digitale* | Cn | *Helgicirrha schulzei* | *Cn* |
|  |  | *Bugula plumosa* | Br |  |  | *Pleuronectes platessa* | Cr | *Praunus flexuosus* | Ar | *Aurelia aurita* | Cn | *Leuckartiara octona* | *Cn* |
|  |  | *Electra sp. LM-2010 ^1^* | Br |  |  | *Raja clavata* | Cr | *Pseudodiaptomus marinus* | Ar | *Clytia hemispherica* | Cn | *Clupea harengus* | *Cr* |
|  |  | *Membranipora membranacea* | Br |  |  | *Echinocyamus pusillus* | Ec | *Temora longicornis* | Ar | *Eucheilota maculata* | Cn | *Pleuronectes platessa* | *Cr* |
|  |  | *Tricellaria occidentalis*³ | Br |  |  | *Ophiura ophiura* | Ec | *Verruca stroemia* | Ar | *Eutima gegenbauri* | Cn | *Raja clavata* | *Cr* |
|  |  | *Bougainvillia muscus ^4^* | Cn |  |  | *Crassostrea gigas* | Mo | *Aglantha digitale* | Cn | *Clupea harengus* | Cr | *Echinocardium cordatum* | *Ec* |
|  |  | *Obelia bidentata* | Cn |  |  | *Loligo forbesii* | Mo | *Aurelia aurita* | Cn | *Pleuronectes platessa* | Cr | *Echinocyamus pusillus* | *Ec* |
|  |  | *Sagartia ornata ^2^* | Cn |  |  | *Sepiola tridens⁰* | Mo | *Clytia hemispherica* | Cn | *Raja clavata* | Cr | *Ophiura ophiura* | *Ec* |
|  |  | *Pomatoschistus minutus* | Cr |  |  | *Electra sp. LM-2010* ^1^ | Br | *Eucheilota maculata* | Cn | *Asterias rubens* | Ec | *Crassostrea gigas* | *Mo* |
|  |  | *Sus scrofa•* | Cr |  |  | *Sagartia ornata* ^2^ | Cn | *Helgicirrha schulzei* | Cn | *Echinocyamus pusillus* | Ec | *Littorina littorea* | *Mo* |
|  |  | *Facelina bostoniensis*^4^ | Mo |  |  | *Sus scrofa•* | Cr | *Leuckartiara octona* | Cn | *Ophiura ophiura* | Ec | *Loligo forbesii* | *Mo* |
|  |  | *Polycera quadrilineata*^5^ | Mo |  |  |  |  | *Obelia dichotoma* | Cn | *Crassostrea gigas* | Mo | *Phaxas pellucidus* | *Mo* |
|  |  | *Lineus bilineatus* ⁰ | Ne |  |  |  |  | *Clupea harengus* | Cr | *Littorina littorea* | Mo | *Sepiola tridens⁰* | *Mo* |
|  |  | *Amphilectus fucorum* | Po |  |  |  |  | *Gadus morhua* | Cr | *Loligo forbesii* | Mo | *Magelona johnstoni* | *An* |
|  |  |  |  |  |  |  |  | *Pleuronectes platessa* | Cr | *Sepiola tridens⁰* | Mo | *Artemia franciscana^7^* | *Ar* |
|  |  |  |  |  |  |  |  | *Raja clavata* | Cr | *Pomatoceros triqueter* | An | *Electra sp. LM-2010 ^1^* | *Br* |
|  |  |  |  |  |  |  |  | *Echinocyamus pusillus* | Ec | *Crisia aculeata*^6^ | Br | *Sagitta setosa* | *Ch* |
|  |  |  |  |  |  |  |  | *Ophiura ophiura* | Ec | *Electra sp. LM-2010* ^1^ | Br | *Hormathia digitata* | *Cn* |
|  |  |  |  |  |  |  |  | *Crassostrea gigas* | Mo | *Tricellaria occidentalis*³ | Br | *Nanomia cara⁰* | *Cn* |
|  |  |  |  |  |  |  |  | *Gibbula cineraria* | Mo | *Sagartia ornata* ^2^ | Cn | *Ophiura albida* | *Ec* |
|  |  |  |  |  |  |  |  | *Loligo forbesii* | Mo | *Ophiura albida* | Ec |  |  |
|  |  |  |  |  |  |  |  | *Ruditapes decussatus** | Mo | *Amphilectus fucorum* | Po |  |  |
|  |  |  |  |  |  |  |  | *Sepiola tridens*⁰ | Mo | *Botryllus schlosseri* | Tu |  |  |
|  |  |  |  |  |  |  |  | *Dodecaceria concharum*⁰ | An |  |  |  |  |
|  |  |  |  |  |  |  |  | *Pomatoceros triqueter* | An |  |  |  |  |
|  |  |  |  |  |  |  |  | *Oithona davisae* | Ar |  |  |  |  |
|  |  |  |  |  |  |  |  | *Rhopalosiphum padi•* | Ar |  |  |  |  |
|  |  |  |  |  |  |  |  | *Crisia aculeata*^6^ | Br |  |  |  |  |
|  |  |  |  |  |  |  |  | *Electra sp. LM-2010* ^1^ | Br |  |  |  |  |
|  |  |  |  |  |  |  |  | *Escharella immersa* | Br |  |  |  |  |
|  |  |  |  |  |  |  |  | *Flustra foliacea* | Br |  |  |  |  |
|  |  |  |  |  |  |  |  | *Membranipora membranacea* | Br |  |  |  |  |
|  |  |  |  |  |  |  |  | *Metridium senile* | Cn |  |  |  |  |
|  |  |  |  |  |  |  |  | *Plumularia setacea⁰* | Cn |  |  |  |  |
|  |  |  |  |  |  |  |  | *Sagartia ornata* ^2^ | Cn |  |  |  |  |
|  |  |  |  |  |  |  |  | *Tubularia indivisa* | Cn |  |  |  |  |
|  |  |  |  |  |  |  |  | *Sus scrofa*• | Cr |  |  |  |  |
|  |  |  |  |  |  |  |  | *Ophiura albida* | Ec |  |  |  |  |
|  |  |  |  |  |  |  |  | *Hiatella sp. K HML-2015* | Mo |  |  |  |  |
|  |  |  |  |  |  |  |  | *Halisarca dujardini*⁰ | Po |  |  |  |  |

* Rare species by Long-term Ecological Research (LTER)

⁰ Not in LTER but plausible in the North Sea

• Terrestrial animal

1 Probably *Electra pilosa,* abundant species in North Sea

2 Not known for North Sea, but members from the same genus, *Sargartia troglodytes* und *S. elegans*

3 *Tricellaria ternata*, same genus known for this area

4 Species, known in the North Sea, but morphologically only identified until genus level

5 Suggestable, but not recorded until now

6 *Crisia ebunea* described in the region. *C. aculeata* is only known in the western parts of the North Sea (Netherlands)

7 Never recorded in the North Sea

**Supplementary Information 4.**

**Lists of the amount of sequences and OTU´s per sample after bioinformatical processing. Given are the identified OTU´s and sequences after taxonomic assignment, inclusive percentages based on the used reference database.**

| **COI mDNA** | | **Obtained** | | **Identified via reference database** | | | | |
| --- | --- | --- | --- | --- | --- | --- | --- | --- |
| **Sample** | | OTU´S | Sequences | **North Sea** | **EMBL** | | | |
| **Station** | **Season** |  |  | OTU´S | OTU´S | Sequences | OTU´S % | Sequences% |
| St. 37 | Spring | 987 | 95947 | 3 | 3 | 65 | 0.3 | 0.1 |
| St. 23 |  | 1487 | 76453 | 14 | 12 | 162 | 0.8 | 0.2 |
| BOX A |  | 649 | 29812 | 17 | 13 | 385 | 2.0 | 1.3 |
| HTR |  | 224 | 12794 | 0 | 0 | 0 | 0.0 | 0.0 |
| Elbe |  | 457 | 10719 | 16 | 16 | 147 | 3.5 | 1.4 |
| Jade |  | 246 | 4987 | 0 | 0 | 0 | 0.0 | 0.0 |
| St. 37 | Summer | 278 | 4749 | 1 | 1 | 4 | 0.4 | 0.1 |
| Station 33 |  | 174 | 5149 | 0 | 0 | 0 | 0.0 | 0.0 |
| Station 23 |  | 357 | 31233 | 4 | 5 | 45 | 1.4 | 0.1 |
| Box A |  | 943 | 41435 | 19 | 23 | 303 | 2.4 | 0.7 |
| HTR |  | 2306 | 126587 | 13 | 16 | 180 | 0.7 | 0.1 |
| Jade |  | 2779 | 158509 | 6 | 6 | 39 | 0.2 | 0.0 |
| St. 34 |  | 2156 | 131016 | 14 | 15 | 360 | 0.7 | 0.3 |
| St. 16 |  | 1391 | 77134 | 6 | 7 | 70 | 0.5 | 0.1 |
| Loreley Bank |  | 2096 | 128553 | 42 | 43 | 641 | 2.1 | 0.5 |
| H. Harbor |  | 2596 | 215817 | 4 | 6 | 69 | 0.2 | 0.0 |
| BOX A | Autumn | 4484 | 469139 | 10 | 18 | 171 | 0.4 | 0.0 |
| HTR |  | 3716 | 457109 |  | 44 | 814 | 1.2 | 0.2 |
| Jade |  | 1846 | 99729 | 3 | 6 | 157 | 0.3 | 0.2 |
| Loreley Bank |  | 2507 | 141193 | 34 | 37 | 800 | 1.5 | 0.6 |
| H.North 01 |  | 3814 | 325558 | 50 | 75 | 1731 | 2.0 | 0.5 |
| H. North 02 |  | 4416 | 458210 | 78 | 59 | 7178 | 1.3 | 1.6 |
| H. West |  | 2647 | 160109 | 42 | 40 | 1384 | 1.5 | 0.9 |
| Artificial Sample |  | 3442 | 436816 |  | 155 | 376468 | 4.5 | 86.2 |

| **18S rDNA** | |  |  |  |  |  |  |
| --- | --- | --- | --- | --- | --- | --- | --- |
| **Sample** | | **Obtained** | | **Identified via reference database** | | | |
| **Station** | **Season** | OTU´S | Sequences | OTU´S | Sequences | OTU´S % | Sequences% |
| St. 37 | Spring | 6796 | 531967 | 317 | 70894 | 4.7 | 13.3 |
| St. 23 |  | 5462 | 282217 | 215 | 16171 | 3.9 | 5.7 |
| BOX A |  | 4523 | 174634 | 195 | 19402 | 4.3 | 11.1 |
| HTR |  | 2557 | 83732 | 166 | 4584 | 6.5 | 5.5 |
| Elbe |  | 9709 | 621666 | 535 | 124905 | 5.5 | 20.1 |
| Jade |  | 4306 | 187192 | 186 | 7658 | 4.3 | 4.1 |
| St. 37 | Summer | 3209 | 100678 | 119 | 4246 | 3.7 | 4.2 |
| Station 33 |  | 2764 | 71311 | 103 | 2696 | 3.7 | 3.8 |
| Station 23 |  | 1904 | 72431 | 75 | 1676 | 3.9 | 2.3 |
| Box A |  | 5132 | 207474 | 248 | 25318 | 4.8 | 12.2 |
| HTR |  | 7160 | 330685 | 281 | 47258 | 3.9 | 14.3 |
| Jade |  | 5910 | 204905 | 178 | 7565 | 3.0 | 3.7 |
| St. 34 |  | 4942 | 168715 | 151 | 7841 | 3.1 | 4.6 |
| St. 16 |  | 3010 | 95338 | 117 | 5330 | 3.9 | 5.6 |
| Loreley Bank |  | 8772 | 355551 | 403 | 45188 | 4.6 | 12.7 |
| H. Harbor |  | 5661 | 228677 | 243 | 46723 | 4.3 | 20.4 |
| BOX A | Autumn | 5117 | 333733 | 169 | 18961 | 3.3 | 5.7 |
| HTR |  | 4108 | 174653 | 175 | 7137 | 4.3 | 4.1 |
| Jade |  | 2637 | 88881 | 119 | 3640 | 4.5 | 4.1 |
| Loreley Bank |  | 7095 | 304008 | 360 | 36869 | 5.1 | 12.1 |
| H.North 01 |  | 3944 | 194257 | 308 | 35082 | 7.8 | 18.1 |
| H. North 02 |  | 3419 | 154892 | 205 | 17567 | 6.0 | 11.3 |
| H. West |  | 3356 | 190949 | 178 | 14305 | 5.3 | 7.5 |
| Artificial Sample |  | 4929 | 501794 | 481 | 386869 | 9.8 | 77.1 |

**Supplementary Information 5.** List of all environmental samples from the stations used for this study and their parallel abiotic data, including depth, voltage (Vbatt), pressure (Press indicating the depth of sampling in m), conductivity (Cond and cap 25), salinity (Salin), the density of seawater (Sigma), and temperature (Temp).

|  |  |  | **Latitude** | **Longitude** | Vbatt | Press | Cond | CAP25 | SALIN | SIGMA | Temp |
| --- | --- | --- | --- | --- | --- | --- | --- | --- | --- | --- | --- |
| **NO.** | **Station** | **Season** | **Dezimal** | **Dezimal** | [Volt] | [dbar] | [mS/cm] | [mS/cm] | [PSU] | [kg/m3] | [°C] |
| 1 | St. 37 | Spring | 55.641 | 4.144 | - | - | - | - | - | - | - |
| 2 | St. 23 |  | 55.007 | 5.357 | - | - | - | - | - | - | - |
| 3 | BOX A |  | 54.369 | 5.357 | - | - | - | - | - | - | - |
| 4 | HTR |  | 54.144 | 7.853 | - | - | - | - | - | - | - |
| 5 | Elbe |  | 54.031 | 8.531 | - | - | - | - | - | - | - |
| 6 | Jade |  | 54.576 | 8.183 | - | - | - | - | - | - | - |
| 7 | St. 37 | Summer | 55.646 | 4.134 | 1.52 | 29.9 | 38.8 | 54.2 | 35.0 | 26.9 | 10.8 |
| 8 | Station 33 |  | 55.336 | 4.404 | 1.52 | 40.6 | 37.3 | 54.5 | 34.9 | 27.2 | 9.2 |
| 9 | Station 23 |  | 55.007 | 5.384 | 1.52 | 33.3 | 40.2 | 54.0 | 35.0 | 26.7 | 12.3 |
| 10 | Box A |  | 54.370 | 7.089 | 1.51 | 29.2 | 42.3 | 52.0 | 33.9 | 25.1 | 15.6 |
| 11 | HTR |  | 54.144 | 7.888 | 1.51 | 23.0 | 42.3 | 50.9 | 33.1 | 24.3 | 16.5 |
| 12 | Jade |  | 53.566 | 8.186 | 1.51 | 6.9 | 46.6 | 49.0 | 32.0 | 21.8 | 22.5 |
| 13 | St. 34 |  | 54.461 | 4.150 | 1.52 | 29.4 | 42.3 | 53.6 | 34.9 | 26.1 | 14.5 |
| 14 | St. 16 |  | 54.666 | 6.623 | 1.52 | 20.6 | 41.4 | 53.0 | 34.4 | 25.8 | 14.0 |
| 15 | Loreley Bank |  | 54.220 | 8.020 | 1.51 | 14.4 | 42.7 | 50.2 | 32.7 | 23.7 | 17.5 |
| 16 | H. Harbor |  | 54.176 | 7.892 | - | - | - | - | - | - | - |
| 17 | BOX A | Autumn | 54.370 | 7.089 | 1.43 | 38.6 | 43.1 | 52.1 | 33.9 | 25.0 | 16.4 |
| 18 | HTR |  | 54.145 | 7.888 | 1.42 | 54.7 | 42.6 | 51.4 | 33.4 | 24.7 | 16.5 |
| 19 | Jade |  | 53.573 | 8.185 | 1.43 | 8.7 | 39.8 | 50.0 | 32.3 | 24.0 | 14.8 |
| 20 | Loreley Bank |  | 54.221 | 8.019 | 1.42 | 14.1 | 42.4 | 51.2 | 33.3 | 24.4 | 16.4 |
| 21 | H.North 01 |  | 54.198 | 7.875 | 1.42 | 13.2 | 42.4 | 51.3 | 33.4 | 24.5 | 16.4 |
| 22 | H. North 02 |  | 54.199 | 7.940 | 1.42 | 13.6 | 42.6 | 51.4 | 33.5 | 24.5 | 16.5 |
| 23 | H. West |  | 54.169 | 7.833 | 1.42 | 44.1 | 42.7 | 51.4 | 33.5 | 24.7 | 16.5 |

**Supplementary Information 6.**

Rarefaction curves of identified metazoan sequences per sample. The analyses based on the results of the EMBL reference database analyses. The identified taxa were sorted and count per sequences (x-axis) based on the taxa identification number (number of taxa, y-axis). The curves are displayed in depending on the three seasons (spring, summer, autumn) and the two gen regions (18S rDNA and COI).


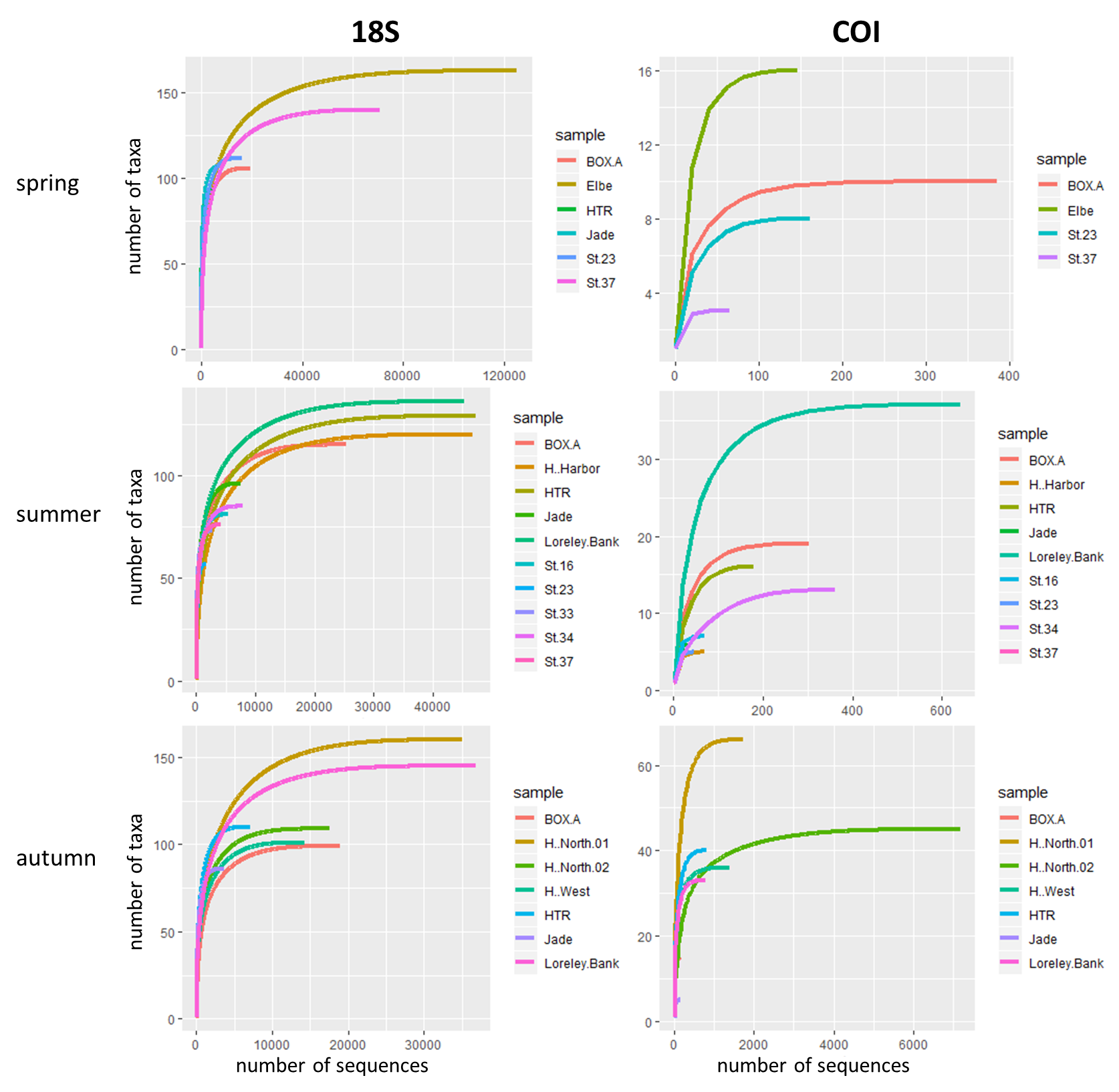

Supplement: Supplementary file 1 — Supplementary Information [file 41598_2018_32917_MOESM1_ESM.doc]
